# Knockdown of ANGPTL2 promotes left ventricular systolic dysfunction by upregulation of NOX4 in mice

**DOI:** 10.3389/fphys.2024.1320065

**Published:** 2024-02-15

**Authors:** Pauline Labbé, Cécile Martel, Yan-Fen Shi, Augusto Montezano, Ying He, Marc-Antoine Gillis, Marie-Ève Higgins, Louis Villeneuve, Rhian Touyz, Jean-Claude Tardif, Nathalie Thorin-Trescases, Eric Thorin

**Affiliations:** ^1^ Montreal Heart Institute, Research Center, Montreal, QC, Canada; ^2^ Department of Pharmacology, Faculty of Medicine, Université de Montréal, Montreal, QC, Canada; ^3^ Research Institute of the McGill University Health Centre, Montreal, QC, Canada; ^4^ Department of Medicine, Faculty of Medicine, Université de Montréal, Montreal, QC, Canada; ^5^ Department of Surgery, Faculty of Medicine, Université de Montréal, Montreal, QC, Canada

**Keywords:** ANGPTL2, NOX4, heart homeostasis, oxidative stress, left ventricular systolic dysfunction

## Abstract

**Background:** Angiopoietin-like 2 (ANGPTL2) is a pro-inflammatory and pro-oxidant circulating protein that predicts and promotes chronic inflammatory diseases such as atherosclerosis in humans. Transgenic murine models demonstrated the deleterious role of ANGPTL2 in vascular diseases, while deletion of ANGPTL2 was protective. The nature of its role in cardiac tissues is, however, less clear. Indeed, in adult mice knocked down (KD) for ANGPTL2, we recently reported a mild left ventricular (LV) dysfunction originating from a congenital aortic valve stenosis, demonstrating that ANGPTL2 is essential to cardiac development and function.

**Hypothesis:** Because we originally demonstrated that the KD of ANGPTL2 protected vascular endothelial function via an upregulation of arterial NOX4, promoting the beneficial production of dilatory H_2_O_2_, we tested the hypothesis that increased cardiac NOX4 could negatively affect cardiac redox and remodeling and contribute to LV dysfunction observed in adult *Angptl2*-KD mice.

**Methods and results:** Cardiac expression and activity of NOX4 were higher in KD mice, promoting higher levels of cardiac H_2_O_2_ when compared to wild-type (WT) mice. Immunofluorescence showed that ANGPTL2 and NOX4 were co-expressed in cardiac cells from WT mice and both proteins co-immunoprecipitated in HEK293 cells, suggesting that ANGPTL2 and NOX4 physically interact. Pressure overload induced by transverse aortic constriction surgery (TAC) promoted LV systolic dysfunction in WT mice but did not further exacerbate the dysfunction in KD mice. Importantly, the severity of LV systolic dysfunction in KD mice (TAC and control SHAM) correlated with cardiac *Nox4* expression. Injection of an adeno-associated virus (AAV9) delivering shRNA targeting cardiac *Nox4* expression fully reversed LV systolic dysfunction in KD-SHAM mice, demonstrating the causal role of NOX4 in cardiac dysfunction in KD mice. Targeting cardiac *Nox4* expression in KD mice also induced an antioxidant response characterized by increased expression of NRF2/KEAP1 and catalase.

**Conclusion:** Together, these data reveal that the absence of ANGPTL2 induces an upregulation of cardiac NOX4 that contributes to oxidative stress and LV dysfunction. By interacting and repressing cardiac NOX4, ANGPTL2 could play a new beneficial role in the maintenance of cardiac redox homeostasis and function.

## 1 Introduction

The search for clinical blood biomarkers in the prognosis and diagnosis of cardiovascular diseases (CVD) is still ongoing. Two decades of research show that elevated circulating levels of pro-inflammatory and pro-oxidative angiopoietin like-2 (ANGPTL2) are independently associated with a risk of coronary artery disease, stroke, diabetes or kidney disease (Thorin et al., 2023). Globally, ANGPTL2 exerts a chronic deleterious inflammatory effect in the vascular system (Kadomatsu et al., 2014; Thorin-Trescases and Thorin, 2014; 2017). In mice, overexpression of ANGPTL2 or exposure to recombinant ANGPTL2 promoted endothelial dysfunction and atherosclerosis (Tabata et al., 2009; Farhat et al., 2013; Horio et al., 2014; Yu et al., 2014). Accordingly, knockout of *Angptl2* preserved endothelial function (Horio et al., 2014) and knockdown (KD) of *Angptl2* increased vascular endothelial stress resistance (Yu et al., 2014; Yu et al., 2015) and delayed atherogenesis (Caland et al., 2019). The effects of ANGPTL2 on the heart are, however, less clear: on the one hand, circulating or cardiac ANGPTL2 levels were found to be independent predictors of the presence of heart failure (Huang et al., 2015; Tian et al., 2016; Tian et al., 2018). In addition, mice overexpressing *Angptl2* in cardiomyocytes exhibited cardiac dysfunction with lower contractility and lower myocardial energy metabolism, while loss of cardiomyocyte-derived *Angptl2* was protective and restored cardiac function (Tian et al., 2016). In murine models of cardiac injury induced by immunotherapy (Horiguchi et al., 2023), septic shock (Li et al., 2023) or the chemotherapeutic drug doxorubicin (Liu et al., 2022), overexpression of ANGPTL2 aggravated cardiac dysfunction and inflammation. ANGPTL2 derived from epicardial adipose tissue could also be involved in fibrotic remodeling of the heart, a substrate for atrial fibrillation (Abe et al., 2018; Kira et al., 2020; Takahashi et al., 2023). Thus, these data suggest that ANGPTL2 contributes to heart diseases and pathological cardiac remodeling (Oike et al., 2017). On the other hand, ANGPTL2 has been proposed to promote physiological remodeling and maintenance of tissue homeostasis (Kadomatsu et al., 2014; Thorin-Trescases et al., 2021); for example, administration of resident cardiac cells expressing ANGPTL2 in rats after myocardial infarction improved LV function and increased neovascularization (Li et al., 2017). We also recently demonstrated that ANGPTL2 plays an essential role in the embryonic development of the heart since KD of *Angptl2* prevented normal aortic valve maturation, leading to congenital aortic valve stenosis (AVS) (Labbé et al., 2022). The AVS observed *in utero* translated into cardiac dysfunction in young and adult *Angptl2*-KD mice, a defect characterized by mild LV dysfunction and cardiac remodeling, but without limiting their lifespan (Labbé et al., 2022). Therefore, the role of ANGPTL2 in cardiac function and remodeling is still controversial and remains to be better characterized.

Left ventricular (LV) cardiac remodeling is an adaptive response to cardiac injuries in order to maintain cardiac output, and is often associated with risk factors such as hypertension, diabetes or aortic stenosis (Opie et al., 2006; Burchfield et al., 2013; Pitoulis and Terracciano, 2020). Inflammatory signaling is strongly involved in cardiac remodeling by the secretion of inflammatory cytokines (Frieler and Mortensen, 2015). In addition, oxidative stress plays a key role in the development of cardiac remodeling (Miao et al., 2022). In particular, NOXs such as NOX2 and NOX4, that are highly expressed in the heart (Lassegue et al., 2012), produce ROS that regulate the level of oxidative stress and contribute to both structural and metabolic myocardial remodeling (Miao et al., 2022). We previously reported that in *Angptl2*-KD mice, vascular endothelial function was preserved via upregulation of NOX4 that produces dilatory H_2_O_2_ while decreasing the activity of NOX2-producing superoxide, preserving eNOS coupling and NO dilatory function (Yu et al., 2015). Since H_2_O_2_ is not only a vasodilatory factor but also a potential hypertrophic factor in cardiomyocytes (Oyama et al., 2009; Qin et al., 2010), we hypothesized that upregulation of NOX4 in cardiac tissues could promote oxidative stress and negatively affect cardiac LV function in *Angptl2*-KD mice.

The aims of the present study were to determine the role of NOXs, particularly NOX4 in cardiac dysfunction and remodeling of adult *Angptl2*-KD mice. We report that i) cardiac protein expression and activity of NOX4 are increased in *Angptl2*-KD compared to wild-type (WT) mice, that ANGPTL2 and NOX4 are co-expressed in cardiac cells of WT mice, and that ANGPTL2 physically interacts with NOX4 in HEK293 cells; ii) the severity of LV systolic dysfunction observed in KD mice correlates with cardiac *Nox4* mRNA expression; iii) *in vivo* cardiac specific reduction of NOX4 expression (with AAV9-shNOX4) fully reverses the deleterious cardiac effects of *Angptl2* KD by activating an antioxidant response characterized by increased cardiac expression of KEAP1/NRF2 and catalase. We propose that the mild cardiac LV dysfunction and remodeling observed in adult *Angptl2*-KD mice could be mediated, at least partially, by an increased cardiac NOX4 activity. Altogether, these data suggest that by interacting and repressing NOX4, ANGPTL2 could play a new beneficial role in the maintenance of cardiac redox homeostasis and function.

## 2 Materials and methods

### 2.1 Animals

All animal experiments were performed in accordance with the “Guide for the Care and Use of Experimental Animals of the Canadian Council on Animal Care,” were approved by the Montreal Heart Institute Ethics Committee (ET 2013-62-3; 2017-62-03) and conducted in compliance with the ARRIVE guidelines.

Generation of the *Angptl2*-KD mouse model was described in (Yu et al., 2014); briefly, it was achieved through a microinjection of a construct generated via retroviral gene trap vectors (developed at Texas A&M Institute for Genomic Medicine) performed in C57Bl/6J mice purchased from The Jackson Laboratory. A β-geo cassette was inserted between bp 5305 and 5390 of the *Angptl2* gene. KD mice were subsequently bred at the Institute for Research in Immunology and Cancer (Montreal, Quebec, Canada); the colony is maintained at the Montreal Heart Institute. All mice used in this study were genotyped by PCR analysis of genomic DNA isolated from ear clips to select both KD and WT animals. Negligible levels of Angptl2 mRNA and protein were confirmed in various tissues including the heart, as previously described (Yu et al., 2014). All mice were kept under standard conditions (24°C; 12:12 h light/dark cycle) and fed a regular diet *ad libitum*.

### 2.2 Echocardiography

Transthoracic echocardiography was performed using an i13L probe (10–14 MHz) and Vivid 7 Dimension system (GE Healthcare Ultrasound, Horten, Norway) in mice sedated by 2% isoflurane. Two-dimensional echocardiography was used to measure dimension of left ventricular (LV) outflow tract and ascending aorta. Peak velocity, peak and mean gradient were measured by sample volume enlarged pulsed wave Doppler. Thickness of LV anterior and posterior wall at end diastole, LV dimension at end diastole and systole, left atrium dimension at end cardiac systole were measured by M-mode echocardiography. LV mass was calculated using a formula previously recommended (Liao et al., 2002). LV fractional shortening and ejection fraction were obtained by a formula available within Vivid 7 system. Trans-mitral flow peak velocity in early filling (E), E deceleration time, deceleration rate, time interval from mitral valve closure to opening were measured by pulsed wave Doppler. Of note, A velocity was not always measurable, resulting in missing values for some diastolic parameters in Tables 1, 2. LV ejection time, stroke volume and cardiac output were measured in LV outflow tract flow and obtained by pulsed wave Doppler. LV iso-volumetric relaxation time was measured by enlarged pulsed wave Doppler. An average of three consecutive cardiac cycles was used for all measurements.

**TABLE 1:** Echo data of *Angptl2*-KD mice and their age-matched WT littermates after TAC surgery.

		WT-SHAM- shSCR (6)	KD-SHAM-shSCR (6)	WT-TAC-shSCR (7)	KD-TAC-shSCR (8)
LV structure	*LV mass (mg)*	117.9 ± 8.3	114.3 ± 16.0	167.9 ± 11.3^b^ ^,^ *	151.3 ± 12.4
	*LV mass/body weight (mg/g)*	4.04 ± 0.32	4.09 ± 0.24	6.24 ± 0.46^b^ ^,^ ***	5.50 ± 0.34^c^ ^,^ *
	*LV anterior wall thickness (mm)*	0.90 ± 0.04	0.85 ± 0.09	1.03 ± 0.04	0.98 ± 0.04
	*LV posterior wall thickness (mm)*	0.72 ± 0.04	0.73 ± 0.06	0.90 ± 0.03^b^ ^,^ **	0.84 ± 0.04
LV systolic function	*Fractional shortening (%)*	36.97 ± 2.80	27.94 ± 1.96^a^ ^,^ *	27.64 ± 2.27^b^ ^,^ *	25.44 ± 2.47
	*Ejection fraction (%)*	72.86 ± 3.54	60.36 ± 3.20	59.85 ± 3.51^b^ ^,^ *	56.06 ± 3.92
	*Cardiac output (mL/min)*	11.98 ± 0.70	11.67 ± 0.69	10.36 ± 1.04	10.36 ± 0.66
	*Stroke volume (µL)*	31.3 ± 1.9	32.2 ± 1.9	26.4 ± 2.7	25.4 ± 1.2^c^ ^,^ *
	*Lateral wall contractility (cm/s)*	2.49 ± 0.08	1.73 ± 0.12^a^ ^,^ ***	1.76 ± 0.12^b^ ^,^ ***	1.58 ± 0.12
	*Septal wall contractility (cm/s)*	2.69 ± 0.04	1.94 ± 0.12^a^ ^,^ **	1.93 ± 0.15^b^ ^,^ **	2.10 ± 0.20
	*LV end-diastolic volume (µL)*	156.2 ± 11.2	168.5 ± 34.3	193.0 ± 23.8	181.1 ± 13.9
	*LV end-systolic volume (µL)*	42.2 ± 5.6	69.2 ± 14.6	81.1 ± 15.9	123.1 ± 36.3
LV diastolic function	*E/A ratio*	1.45 ± 0.06 (3)	1.62 ± 0.09 (3)	1.16 (1)	3.61 ± 1.87 (3)
	*E/E′ ratio*	29.24 ± 2.34 (5)	32.64 ± 4.08 (5)	29.86 (1)	40.95± 2.02 (4)
	*E velocity (cm/s)*	90.34 ± 3.12	80.25 ± 8.37 (5)	87.36 ± 6.52	93.95 ± 5.00 (7)
	*A velocity (cm/s)*	58.60 ± 1.70 (3)	48.92 ± 9.62 (3)	52.31 (1)	40.77 ± 14.67 (3)
	*E deceleration time (ms)*	35.71 ± 2.44	38.26 ± 5.02 (5)	32.25 ± 2.55	28.76 ± 1.99 (7)
	*E deceleration rate (m/s* ^*2*^ *)*	25.85 ± 1.55	21.99 ± 2.68 (5)	28.22 ± 3.06	34.01 ± 3.83 (7)^c^ ^,^ *

Data are mean ± SEM, of (n) WT and KD mice. *: *p* < 0.05; **: *p* < 0.01; ***: *p* < 0.001; determined with 2-way ANOVA, Bonferroni post-hoc test.

^a^ WT-SHAM-shSCR, vs. KD-SHAM-shSCR.

^b^ WT-SHAM-shSCR, vs. WT-TAC-shSCR.

^c^ KD-SHAM-shSCR, vs. KD-TAC-shSCR.

**TABLE 2:** Echo data of Angptl2-KD mice and their age-matched WT littermates after targeting cardiac NOX4 expression.

		WT-SHAM- shSCR (6)	KD-SHAM-shSCR (6)	WT-SHAM-shNOX4 (8)	KD-SHAM-shNOX4 (7)
LV structure	*LV mass (mg)*	117.9 ± 8.3	114.3 ± 16.0	113.3 ± 6.4	122.1 ± 13.8
	*LV mass/body weight (mg/g)*	4.04 ± 0.32	4.09 ± 0.24	4.00 ± 0.30	4.41 ± 0.35
	*LV anterior wall thickness (mm)*	0.90 ± 0.04	0.85 ± 0.09	0.86 ± 0.02	0.86 ± 0.03
	*LV posterior wall thickness (mm)*	0.72 ± 0.04	0.73 ± 0.06	0.77 ± 0.04	0.83 ± 0.03
LV systolic function	*Fractional shortening (%)*	36.97 ± 2.80	27.94 ± 1.96^a^ ^,^ *	35.30 ± 1.78	37.56 ± 1.87^c^ ^,^ **
	*Ejection fraction (%)*	72.86 ± 3.54	60.36 ± 3.20^a^ ^,^ *	71.12 ± 2.17	73.94 ± 2.30^c^ ^,^ **
	*Cardiac output (mL/min)*	11.98 ± 0.70	11.67 ± 0.69	9.99 ± 0.34^b^ ^,^ *	11.20 ± 0.61
	*Stroke volume (µL)*	31.3 ± 1.9	32.2 ± 1.9	28.0 ± 1.2	29.9 ± 2.6
	*Lateral wall contractility (cm/s)*	2.49 ± 0.08	1.73 ± 0.12 ^a^ ^,^ ****	2.38 ± 0.05	2.25 ± 0.11^c^ ^,^ **
	*Septal wall contractility (cm/s)*	2.69 ± 0.04	1.94 ± 0.12 ^a^ ^,^ ***	2.57 ± 0.11	2.55 ± 0.12^c^ ^,^ **
	*LV end-diastolic volume (µL)*	156.2 ± 11.2	168.5 ± 34.3	145.8 ± 11.3	147.4 ± 18.8
	*LV end-systolic volume (µL)*	42.2 ± 5.6	69.2 ± 14.6	42.9 ± 5.6	40.7 ± 9.0
LV diastolic function	*E/A ratio*	1.45 ± 0.06 (3)	1.62 ± 0.09 (3)	1.43 ± 0.05 (5)	2.27 ± 0.73 (4)
	*E/E′ ratio*	29.24 ± 2.34 (5)	32.64 ± 4.08 (5)	30.93 ± 2.97 (5)	30.44 ± 1.65 (6)
	*E velocity (cm/s)*	90.34 ± 3.12	80.25 ± 8.37 (5)	77.23 ± 2.79	88.35 ± 3.44
	*A velocity (cm/s)*	58.60 ± 1.70 (3)	48.92 ± 9.62 (3)	52.75 ± 2.50 (5)	45.63 ± 8.22 (4)
	*E deceleration time (ms)*	35.71 ± 2.44	38.26 ± 5.02 (5)	32.59 ± 3.55	31.40 ± 3.09
	*E deceleration rate (m/s* ^*2*^ *)*	25.85 ± 1.55	21.99 ± 2.68 (5)	25.78 ± 2.72	29.35 ± 2.06

Data are mean ± SEM, of (n) WT and KD mice. *: *p* < 0.05; **: *p* < 0.01; ***: *p* < 0.001; ****: *p* < 0.0001; determined with 2-way ANOVA, Bonferroni post-hoc test.

^a^ WT-SHAM-shSCR, vs. KD-SHAM-shSCR.

^b^ WT-SHAM-shSCR, vs. WT-SHAM-shNOX4.

^c^ KD-SHAM-shSCR, vs. KD-SHAM-shNOX4.

### 2.3 TAC surgery

5.5-month-old WT and KD mice were subjected to TAC or sham surgery, for 6 weeks. Mice were anesthetized with 2.5% isoflurane and intubated. After intercostal incision, the aorta was partially ligated between the innominate and the left common carotid artery with a 7-0 silk using a 27-gauge guide, leading to 60% aortic constriction. The guide was rapidly removed, the chest closed and mice extubated. The sham procedure was identical except from the aorta ligation. Mice received analgesia via intra-peritoneal injection of buprenorphine (0.1 mg/kg) before surgery and 2 doses after surgery with ketoprofen (Anafen^®^; 20 mg/kg) over 24 h. Mice were sacrificed 6 weeks after TAC surgery.

### 2.4 Sample collection

At the age of 7 months, animals were exsanguinated under anesthesia and organs were collected. Hearts were either, frozen in liquid nitrogen and stored at −80°C, or fixed in formalin for histological studies.

### 2.5 Histological studies

Hearts were embedded in paraffin and transverse sections were stained by Hematoxylin/Eosin (H&E) or Masson’s trichrome and were imaged with an Olympus BX45 microscope. 40X magnitude pictures were analyzed (3 per individual) and fibrosis was quantified with Image Pro V7 (Media Cybernetics).

### 2.6 Immunofluorescence

Fixed tissues (longitudinally frozen heart sections from 3-month old mice) were incubated with 1:50 diluted goat anti-mAngptl2 (AF1444, R&D Systems); 1:100 diluted rat anti-CD31 (AF3628, R&D Systems); 1:100 diluted rabbit anti-Vimentin (ab137321, Abcam); 1:100 diluted rabbit anti-alpha smooth muscle actin (α-SMA; ab5694, Abcam); 1:100 diluted rabbit anti-NOX4 (NB110-58849, Novus Biologicals); 1:500 diluted secondary antibodies Alexa fluor-647 anti-rabbit (A31573, ThermoFisher); Alexa fluor-488 anti-rat (A21208, ThermoFisher) and Alexa fluor-555 anti-goat (A21432, ThermoFisher). DNA counterstaining was performed by incubating fixed tissues with 1:600 diluted DAPI (D1306, ThermoFisher). Tissue cryosections were then mounted on glass slides (Superfrost Plus, Fisher Scientific) and dried overnight. Images were acquired with a LSM 710 confocal microscope (Zeiss) using Plan APO 40X/1.3 oil DIC and Plan APO 63X/1.4 oil DIC objectives. Images are maximum intensity projections created with Z-stack (0.5 µm Z-steps).

### 2.7 Immunoblotting

Whole hearts of mice were pulverized into powder, and lysed in 100 mM NaCl, 50 mM Tris pH7.5, 20 mM β-glycerophosphate, 20 mM NaF, 1% NP-40, 1 mM PMSF, 5 mM EDTA, 10 mM EGTA, 1 mM Na_3_VO_4_, 1X protease inhibitor cocktail (cOmplete™ Mini, Roche) and clarified by centrifugation (16,000 g for 20 min at 4°C). Protein concentrations were measured using the Protein Assay Dye Reagent Concentrate (#500-0006, Bio-Rad). Aliquots containing 40–50 μg of protein were denatured for 5 min at 95°C in the presence of 10 mM DTT, separated by SDS-PAGE and transferred to nitrocellulose membrane. Immunoblots were probed with antibodies against NOX4 (1:1000, ab133303, Abcam) or NOX4 (1:500, NB110-58849, Novus Biologicals), ANGPTL2 (1:1000, AF 2084, R&D Systems), phospho (Thr180/Tyr182)-p38 MAPK (1:500, 9211, Cell Signaling), p38 MAPK (1:1000, 8690, Cell Signaling), NRF2 (1:500, sc-722, Santa Cruz), KEAP1 (1:500, sc-15246, Santa Cruz), Catalase (1:1000, #219010, Calbiochem^®^, Sigma-Aldrich), p22phox (1:500, sc-20781, Santa Cruz), NOX2 (1:500, ab43801, Abcam) and GAPDH (1:1000, AM4300, Ambion). Chemiluminescence was used to detect protein expression (Western Lightning Plus ECL, Perkin Elmer).

### 2.8 Measurement of NADPH-dependent ROS production

Cardiac production of superoxide (O_2_^−^) was measured by lucigenin-enhanced chemiluminescence assay, with lucigenin as the electron acceptor and NADPH as the substrate as previously described (Briones et al., 2011). Ventricles (10 mg) were homogenized in assay buffer (50 mmol/L of KH_2_PO_4_, 1 mmol/L of EGTA, and 150 mmol/L of sucrose, pH 7.4). The assay was performed with 50 µL of sample, 1.25 µL of lucigenin (5 μmol/L), 25 µL of NADPH (0.1 mmol/L) and assay buffer to a total volume of 250 µL. Luminescence was measured for 30 cycles of 1 s each by a luminometer (Orion II/MPL4 microplate luminometer, Berthold Detection Systems, Germany). Basal readings were obtained prior to the addition of NADPH to the assay. The reaction was initiated by the addition of the substrate. Basal and buffer blank values were subtracted from the NADPH-derived luminescence. Superoxide production was expressed as relative luminescence unit (RLU)/µg/μL protein. Inhibitors of NOX1 [ML 171 (Tocris; 4653)] and NOX2 [gp91 ds-tat (ANASPEC; AS-63818)] were used in the lucigenin assay to inhibit NOX1 and NOX2, but not NOX4. Tissue homogenates were treated with 1 µM gp91 ds-tat and 1 μM ML 171 (diluted in 0.9% saline with 0.01 N acetic acid) for 30 min at 37°C before luminescence measurement.

### 2.9 Measurement of cardiac H_2_O_2_ production

Total cardiac hydrogen peroxide (H_2_O_2_) levels were assessed in 20 µL of tissue homogenate with PeroxiDetect kit (Sigma-Aldrich; PD1) according to the manufacturer’s instructions. Absorbance was read at 560 nm by a spectrophotometer. H_2_O_2_ levels were expressed as μmol/μg protein.

### 2.10 Real-time qPCR

Total RNA was extracted from frozen ventricles using RNeasy mini-kit (Qiagen) and reverse transcription was performed on 1 µg RNA using the Moloney murine leukemia virus reverse transcriptase (Invitrogen) according to the manufacturer’s instructions. Quantitative polymerase chain reaction (qPCR) was performed using 2X Platinum SYBR Green qPCRSuperMix-UDG (Invitrogen) and MX3005p qPCR System cycler (Stratagene) according to manufacturer’s protocol. Primers of target genes were designed using the Clone Manager software and were the following: NOX4-Fwd: 5′-ATC​CTT​TTA​CCT​ATG​TGC​CGG-3′; NOX4-Rev: 5′-CTT​TCT​GGG​ATC​CTC​ATT​CTG​G-3′; NOX2-Fwd: 5′-AGT​GCC​CAG​TAC​CAA​AGT​TC-3′; NOX2-Rev: 5′-GTC​CCA​CCT​CCA​TCT​TGA​ATC-3′; Cyclophilin A-Fwd: 5′-CCG​ATG​ACG​AGC​CCT​TGG-3′; Cyclophilin A-Rev: 5′-GCC​GCC​AGT​GCC​ATT​ATG-3′. ΔΔCT were determined with MxPro v4.10 software using cyclophilin A as a housekeeping gene.

### 2.11 Plasmid production and AAV-9 purification

Adeno-associated virus serotype 9 (AAV9) producing small hairpin RNA (shRNA) against mouse NOX4 (shNOX4) or scrambled sequence (shSCR) were generated according to a protocol adapted from a previous study (Caland et al., 2019). Briefly, single stranded cDNAs containing different shRNA flanked by HindIII and BamHI sites and 2 linkers were purchased from IDT and amplified by PCR using linker primers (fwd: 5′ TAA​CCC​TCA​CTA​AAG​GGA​CTC, rev: 5′ TAA​TAC​GAC​TCA​CTA​TAG​GGC​TC). Double stranded amplicons were digested at the 2 restriction sites, gel purified and ligated in pU6 ITR plasmid (a kind gift from Dr N. Bousette, Montreal, Canada) followed by transformation in Stbl3 bacteria. All used clones were confirmed by sequencing.

shSCR sequence (Addgene plasmid #1864) was:

5′TAA​CCC​TCA​CTA​AAG​GGA​CTC​AAG​CTT​TTC​CAA​AAA​ACC​TAA​GGT​TAA​GTC​GCC​CTC​GCT​CTT​GAC​GAG​GGC​GAC​TTA​ACC​TTA​GGC​GGG​ATC​CAT​CGA​GCC​CTA​TAG​TGA​GTC​GTA​TTA

shNOX4 sequence was based on the siRNA sequence previously published (Peterson et al., 2009):

5′TAA​CCC​TCA​CTA​AAG​GGA​CTC​AAG​CTT​TTC​CAA​AAA​AGG​AAC​AAG​TGC​AAT​TTC​TAA​GCT​CTT​GAC​TTA​GAA​ATT​GCA​CTT​GTT​CCC​GGG​ATC​CAT​CGA​GCC​CTA​TAG​TGA​GTC​GTA​TTA.

AAV9 vectors with wild-type capsids were generated by co-transfection with the helper plasmid pDG-9 in HEK 293T cells using the polyethylenimine protocol (Caland et al., 2019). 72 h after transfection, cells were harvested and AAV9 were isolated, purified and dialyzed using iodixanol gradient technique as previously described (Toko et al., 2013). Virus titration was assessed by qPCR with the following primers (fwd: 5′ TGCCTTCCTTGACCCT, rev: 5′ CCTTGCTGTCCTGCCC). AAV9-shNOX4 or AAV9-shSCR (1 × 10^11^ copies) was injected intravenously, once, in the tail vein at the age of 6 months and the mice were sacrificed at the age of 7 months.

### 2.12 Co-immunoprecipitation

HEK293 cells transfected with a pBlueScript SK (+) vector (Addgene #212205) containing the target DNA sequence for human Angptl2—subcloned using EcoRI/HindIII restriction sites between the T3 and T7 promoters—were lysed in: 100 mM NaCl, 50 mM Tris pH 7.5, 50 mM NaF, 1% NP-40, 1 mM PMSF, 1X protease inhibitor cocktail (complete™ Mini, Roche) and clarified by centrifugation (21,000 g for 10 min at 4°C). To immunoprecipitate endogenous NOX4, 0.5–1.0 mg of cell lysates was incubated with 3 μg of anti-NOX4 antibody (ab133303) cross linked to 12.5 μL of protein A conjugated magnetic beads (Dynabeads, Invitrogen). hANGPTL2 was immunoprecipitated using Pierce anti-HA magnetic beads (20 μL) (Life Technologies). Lysates were incubated for 2 h at 4°C, the immunoprecipitates were washed three times with lysis buffer and eluted in 1× Laemmli sample buffer for 10 min at 95°C. Eluted proteins were denatured for 5 min at 95°C in the presence of 10 mM DTT, separated by SDS-PAGE and transferred to nitrocellulose membrane. Immunoblots were probed with appropriate antibodies, and chemiluminescence was used to detect protein expression (Western Lightning Plus ECL, Perkin Elmer).

### 2.13 Statistics

Data are presented as means ± SEM, with n indicating the number of mice. Mann–Whitney U test was used to assess differences between WT and KD mice, and between KD mice subjected to SHAM surgery injected with AAV9-shSCR or AAV9-shNOX4. A two-way ANOVA with Bonferroni’s multiple comparisons test was performed to compare WT and KD mice treated with AAV9-shSCR or AAV9-shNOX4. Spearman correlation analyses were performed to correlate the mRNA levels of *Nox4* and the severity of cardiac dysfunction. A *p*-value < 0.05 was considered statistically significant. All statistics were performed using GraphPad Prism 10 Software for macOS.

## 3 Results

### 3.1 *Angptl2*-KD mice exhibit mild LV systolic dysfunction and increased cardiac NOX4 expression and activity

When compared to age-matched WT mice, we previously reported that 7-month-old KD mice developed mild LV systolic dysfunction characterized by a decrease in both LV fractional shortening (LVFS) and ejection fraction (LVEF) (Labbé et al., 2022). At that age, male and female KD mice also exhibited a smaller body weight and a similar heart weight, leading to a higher heart mass/body weight ratio (Supplementary Figure S1A) associated with a trend (*p* = 0.0513) in increased cardiac fibrosis (Supplementary Figure S1B). These data suggest that *Angptl2*-KD mice are characterized by mild LV systolic dysfunction and by mild LV remodeling.

**FIGURE 1:**
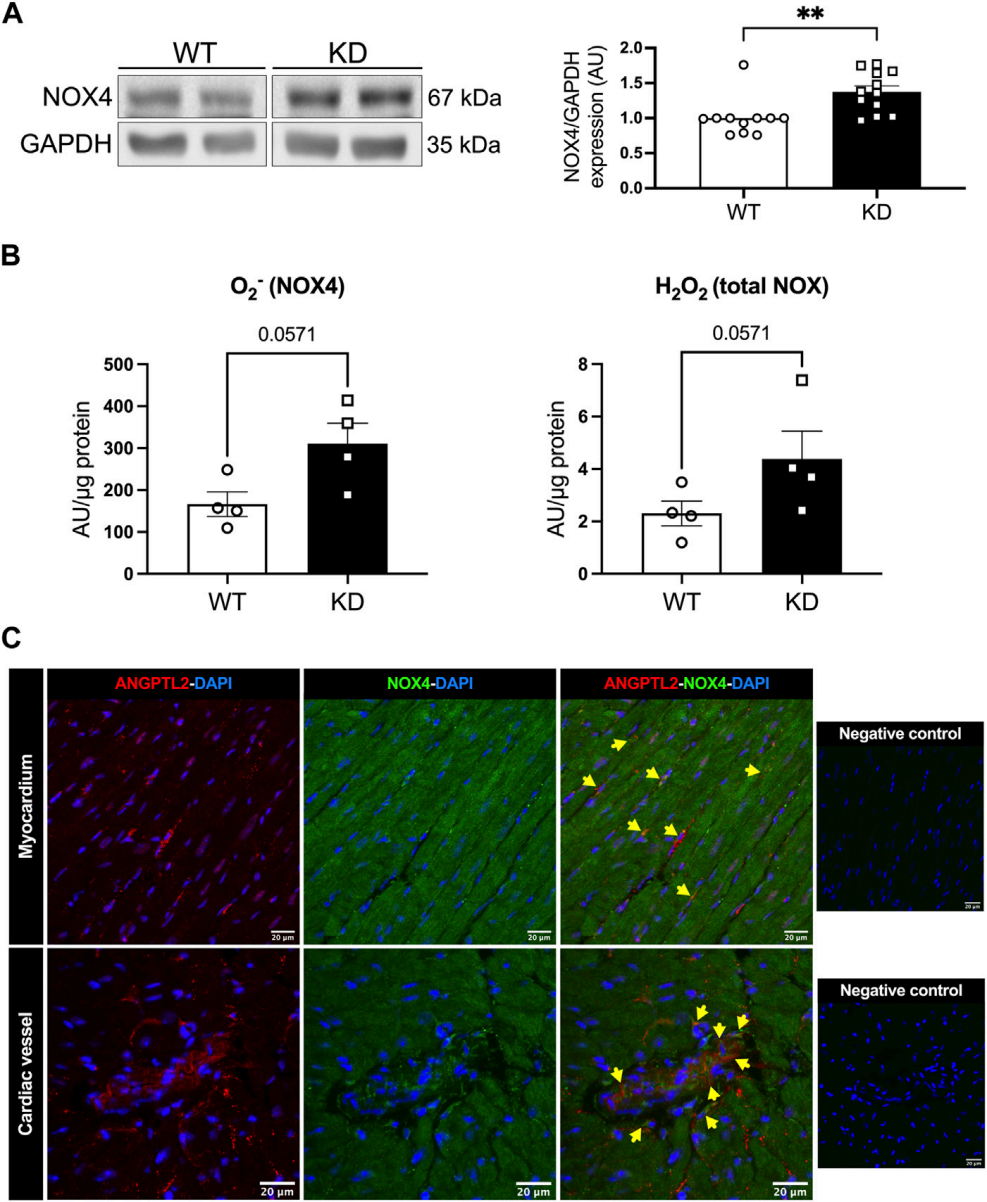
*Angptl2*-KD mice exhibit increased cardiac NOX4 expression and activity. ANGPTL2 and NOX4 are co-expressed in the heart. **(A)** Protein expression of NOX4, and GAPDH as loading control, detected by Western Blot in hearts from KD mice compared to WT mice. Data are mean ± SEM of *n* = 11 male WT mice and *n* = 12 male KD mice. **: *p* < 0.01 vs. WT mice determined with Mann-Whitney U test. **(B)** Cardiac ROS generation was measured by lucigenin-enhanced chemiluminescence assay. Superoxide (O_2_^−^) production was expressed as relative luminescence unit (AU)/µg protein. NOX1 inhibitor ML 171 and NOX2 inhibitor gp91 ds-tat were used to reveal NOX4 activity. Cardiac hydrogen peroxide (H_2_O_2_) production was detected spectrophotometrically in cardiac tissue and was expressed as arbitrary unit (AU)/µg protein. Data are mean ± SEM of *n* = 4 mice per genotype. *p* = 0.0571 vs. WT mice determined by Mann-Whitney U test. **(C)** Representative images of ANGPTL2 and NOX4 protein expression by immunofluorescence in heart sections from 3-month old WT mice. ANGPTL2 and NOX4 are co-expressed (indicated by yellow arrows) in some cells in the myocardium and in cardiac vessels. DAPI staining was used to visualize cell nuclei. Images are representative of *n* = 3 independent experiments.

We then investigated whether NOX4 contributes to cardiac dysfunction in KD mice. Protein expression levels of NOX4 were increased in hearts of KD mice compared to WT mice (+38%; *p* < 0.01; Figure 1A). NADPH-dependent generation of superoxide (O_2_^−^), in the presence of NOX1 and NOX2 inhibitors, was higher in hearts of KD mice versus controls (+86%, with a significance level of *p* = 0.0571; Figure 1B), suggesting increased NOX4 activity. In addition, total hydrogen peroxide (H_2_O_2_) cardiac levels tended (*p* = 0.0571) to be higher in KD mice (+87%; Figure 1B). Taken together, these data indicate that cardiac NOX4 expression and activity are higher in KD mice, promoting oxidative stress.

The spatial expression of ANGPTL2 and NOX4 was then assessed by immunofluorescence in cardiac histological sections from WT mice. In myocardium and cardiac vessels, ANGPTL2 was heterogeneously expressed in some cells, whereas NOX4 expression was ubiquitous as previously described (Bedard and Krause, 2007) (Figure 1C). ANGPTL2 and NOX4 appeared to be co-expressed in some cells both in the myocardium and in cardiac vessels (Figure 1C).

**FIGURE 2:**
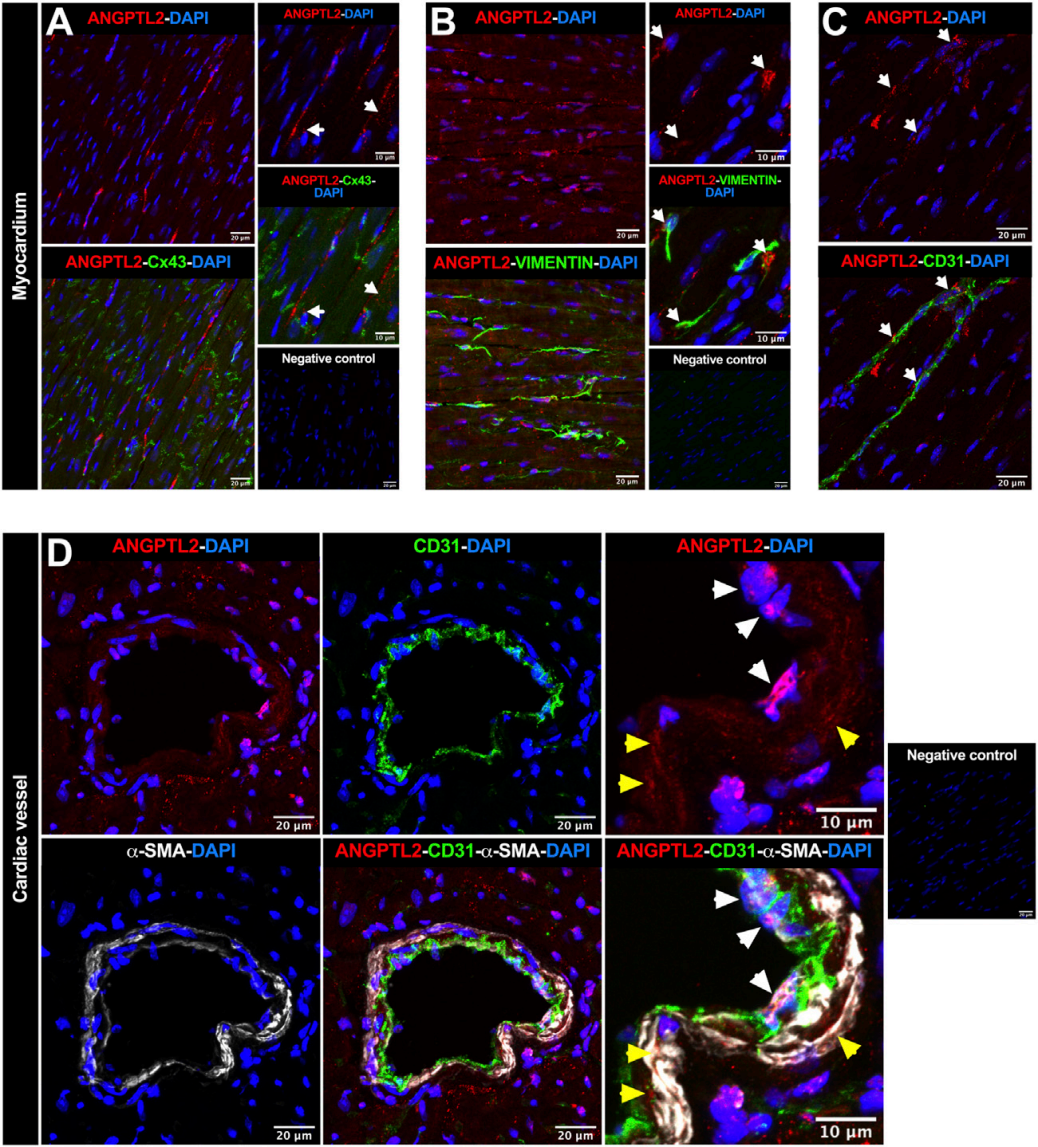
Spatial expression of ANGPTL2 in the myocardium and cardiac vessels. **(A)** Representative images of ANGPTL2 and Cx43 protein expression by immunofluorescence in heart sections from 3-month old WT mice. ANGPTL2 is expressed in some cardiomyocytes delimited with Cx43 (indicated by white arrows). **(B)** Representative images of ANGPTL2 and vimentin protein expression by immunofluorescence in heart sections from 3-month-old WT mice. ANGPTL2 is expressed in vimentin-positive cardiac fibroblasts (indicated by white arrows). **(C)** Representative images of ANGPTL2 and CD31 protein expression by immunofluorescence in heart sections from 3-month-old WT mice. ANGPTL2 is expressed in CD31-positive endothelial cells (indicated by white arrows). **(D)** Representative images of ANGPTL2, CD31 and alpha-smooth muscle actin (α-SMA) protein expression by immunofluorescence in heart sections containing cardiac blood vessel from 3-month-old WT mice. Higher magnification shows that ANGPTL2 is co-expressed with both CD31-positive cells (indicated by white arrowheads) and α-SMA-positive cells (indicated by yellow arrowheads). DAPI staining was used to visualize cell nuclei. Images are representative of *n* = 3 independent experiments.

To investigate in which cell types ANGPTL2 and NOX4 may be involved in mutual signaling pathways in the heart, we focused on the spatial expression of ANGPTL2, as NOX4 expression is ubiquitous. In myocardium, ANGPTL2 was detected in some cardiac myocytes—defined by the gap junction protein connexin 43 (Cx43)—and exhibited a punctiform expression pattern (Figure 2A). ANGPTL2 was mostly co-expressed with vimentin-positive cardiac fibroblasts (Figure 2B) and CD31-positive endothelial cells (Figure 2C). In cardiac vessels, ANGPTL2 appeared to be expressed both in CD31-positive endothelial cells and in alpha-smooth muscle actin (α-SMA)-positive cells (Figure 2D). These data suggest that ANGPTL2 and NOX4 could be co-expressed in cardiac fibroblasts, but also in cardiomyocytes, endothelial and vascular smooth muscle cells.

**FIGURE 3:**
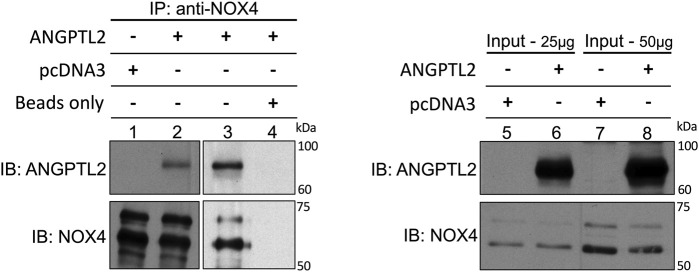
ANGPTL2 and NOX4 physically interact in HEK293 cells. HEK293 cells were transfected with the empty pcDNA3 vector (lane 1) or a pcDNA3 vector containing the human isoform of ANGPTL2 (lane 2). ANGPTL2 was readily co-immunoprecipitated with NOX4 using anti-NOX4 antibody (upper blot, lane 2). The specificity of the co-IP was partly attested by the absence of immunoprecipitation of pcDNA3 by anti-NOX4 antibody (lane 1). ANGPTL2 was repeatedly co-immunoprecipitated with NOX4 using NOX4 antibody (lane 3). A control IP confirmed that ANGPTL2 protein did not stick to magnetic beads (lane 4). Immunoblots of input controls (25–50 µg of total lysate) for ANGPTL2 and NOX4 are also shown (lanes 5–8). Images are representative of *n* = 3 independent experiments. IP: immunoprecipitation; IB: immunoblot.

Finally, to decipher whether ANGPTL2 may physically interact with NOX4, we performed immunoprecipitations of endogenous NOX4 in HEK293 cells transfected with a plasmid encoding for the human isoform of ANGPTL2. We validated that NOX4 co-immunoprecipitates with ANGPTL2 (Figure 3, lanes 2-3 and 5–8). Control immunoprecipitations confirmed that the anti-NOX4 antibody did not co-immunoprecipitate in a condition transfected with the pcDNA3 vector only (Figure 3, lane 1) and that ANGPTL2 protein did not stick to magnetic beads (Figure 3, lane 4). Hence, ANGPTL2 and NOX4 are co-expressed in both cardiac and vascular cells and ANGPTL2 protein can physically interact with NOX4.

### 3.2 Targeting cardiac NOX4 fully reverses LV systolic dysfunction in *Angptl2*-KD mice

**FIGURE 4:**
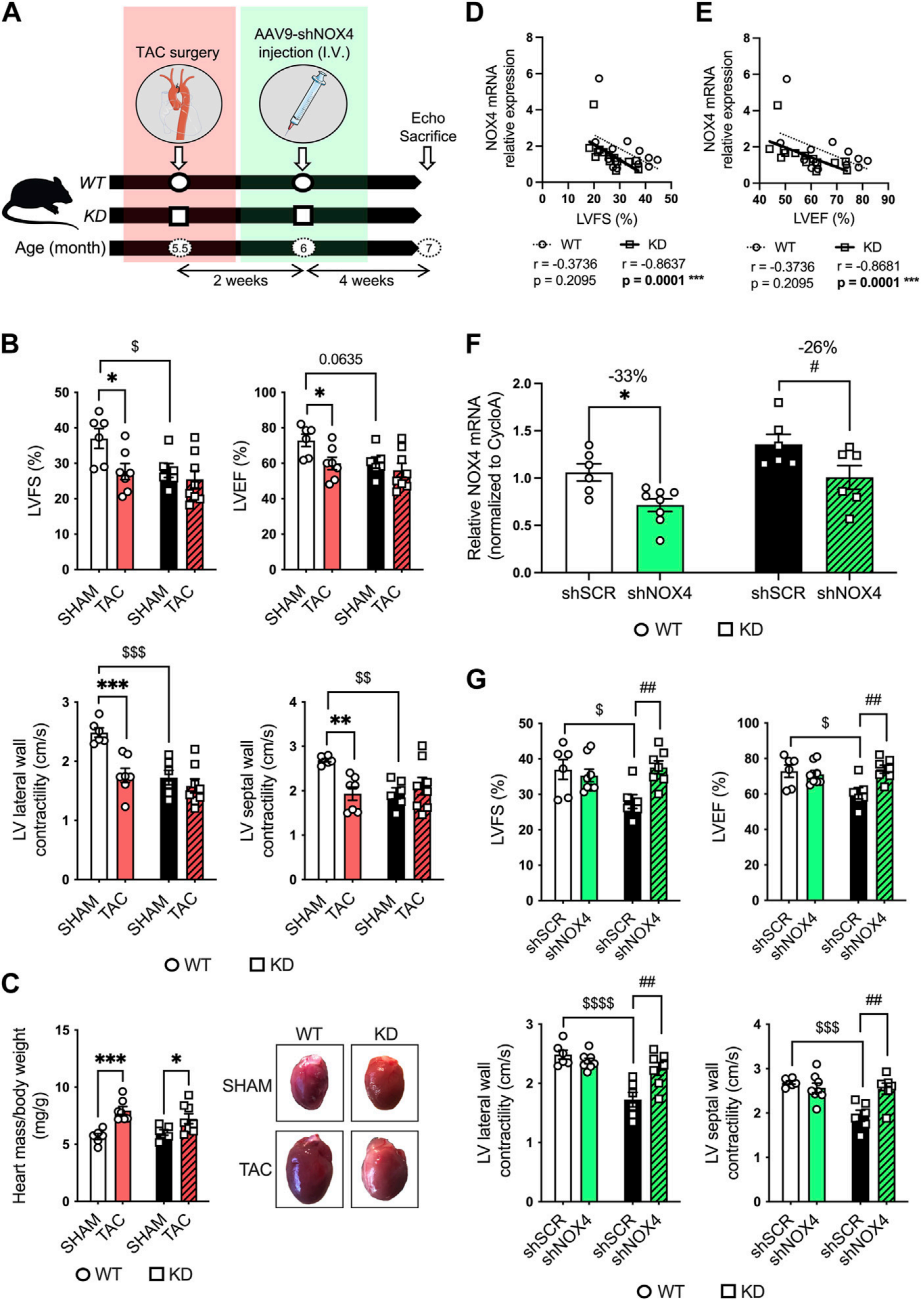
Targeting cardiac NOX4 fully reverses LV systolic dysfunction in *Angptl2*-KD mice. **(A)** Experimental protocol and timeline for TAC (or control SHAM) surgery and injection of AAV9-shNOX4 (or control shSCR) in WT and KD mice. Parts of the figure were drawn by using pictures from Servier Medical Art. Servier Medical Art by Servier is licensed under a Creative Commons Attribution 3.0 Unported License. **(B)** Echocardiographic measurements of LVFS, LVEF, and LV lateral and septal wall contractility in WT and KD mice subjected to SHAM or TAC surgery. LVFS: left ventricular fractional shortening; LVEF: left ventricular ejection fraction. Data are mean ± SEM of n = 6 WT mice and *n* = 6 KD mice in the SHAM group, and *n* = 7 WT mice and *n* = 8 KD mice in the TAC group. *: *p* < 0.05 vs. WT mice. $: *p* < 0.05 vs. SHAM; determined with 2-way ANOVA, Bonferroni post-hoc test. **(C)** Heart weight/body weight ratio in KD mice subjected to SHAM or TAC surgery, and representative images of whole hearts from WT and KD mice (right panel). Data are mean ± SEM of *n* = 6 WT mice and *n* = 6 KD mice in the SHAM group, and *n* = 7 WT mice and *n* = 8 KD mice in the TAC group. *: *p* < 0.05 vs. SHAM; determined with 2-way ANOVA, Bonferroni post-hoc test. **(D,E)** Linear correlations between cardiac NOX4 relative mRNA levels and **(D)** fractional shortening and **(E)** ejection fraction in WT and KD mice subjected or not (SHAM) to TAC surgery. Data are means ± SEM of *n* = 13 WT mice and *n* = 14 KD mice. *: *p* < 0.05 determined with Spearman correlation tests. **(F)** Relative NOX4 mRNA expression determined by quantitative RT-qPCR in hearts from WT and KD mice subjected to SHAM surgery and injected with AAV9-shSCR or AAV9-shNOX4. Data are mean ± SEM of *n* = 6 WT mice and *n* = 6 KD mice in the AAV9-shSCR group, and *n* = 8 WT mice and *n* = 6 KD mice in the AAV9-shNOX4 group. *: *p* < 0.05 WT-shSCR vs. WT-shNOX4 mice. #: *p* < 0.05 KD-shSCR vs. KD-shNOX4; determined with 2-way ANOVA, Bonferroni post-hoc test. **(G)** Echocardiographic measurements of LVFS, LVEF, and LV lateral and septal wall contractility in WT and KD mice subjected to SHAM surgery and injected with AAV9-shSCR or AAV9-shNOX4. Data are mean ± SEM of *n* = 6 WT mice and *n* = 6 KD mice in the AAV9-shSCR group, and *n* = 8 WT mice and *n* = 7 KD mice in the AAV9-shNOX4 group. *: *p* < 0.05 WT-shSCR vs. WT-shNOX4 mice. $: *p* < 0.05 WT-shSCR vs. KD-shSCR mice. #: *p* < 0.05 KD-shSCR vs. KD-shNOX4; determined with 2-way ANOVA, Bonferroni post-hoc test.

To further investigate LV dysfunction in KD mice, mice were subjected to transaortic constriction (TAC) surgery. To demonstrate the role of NOX4 in LV dysfunction in KD mice, 5.5-month-old male and female KD mice and age- and sex-matched WT littermates were first subjected to TAC surgery (or control: SHAM); 2 weeks later, mice received a single intravenous injection of AAV9-shRNA targeting cardiac expression of *Nox4* (shNOX4) or control AAV9-shScramble (shSCR) (Figure 4A). Six weeks post-TAC, that include 4 weeks of NOX4 targeting, echocardiography was performed, and mice were sacrificed when they reached the age of 7 months (Figure 4A).

Compared to WT mice, 7-month old SHAM-KD mice exhibited AVS characterized by increases (*p* < 0.01) in transaortic peak velocity (170 ± 33 vs. 94 ± 5 cm/s, *n* = 6; Supplementary Table S1) and in mean pressure gradient (8 ± 3 vs. 2.0 ± 0.2 mm Hg, *n* = 6; Supplementary Table S1), as previously described in younger mice (Labbé et al., 2022); SHAM-KD mice also exhibited mild LV systolic dysfunction characterized by a significant decrease (*p* < 0.05) in LVFS (Figure 4B; Table 1), a strong trend (*p* = 0.0635) towards a reduction in LVEF (Figure 4B; Table 1), and a significant decrease (*p* < 0.01) in LV lateral and septal wall contractility (Figure 4B; Table 1). These data confirm that adult SHAM-KD mice develop mild LV systolic dysfunction (Labbé et al., 2022).

In both WT and KD mice, TAC surgery equally reduced aortic diameter at the area of banding and increased aortic peak velocity, peak gradient and mean gradient (TAC vs. SHAM, *p* < 0.01; Supplementary Table S2). In addition, TAC significantly increased the cardiac hypertrophy index LV mass/body weight ratio for both KD (TAC: 7.25 ± 0.44 vs. SHAM: 6.05 ± 0.24 mg/g, *p* < 0.05; Figure 4C) and WT mice (TAC: 7.97 ± 0.35 vs. SHAM: 5.68 ± 0.25 mg/g, *p* < 0.001; Figure 4C). The effects of TAC surgery on heart structure of WT and KD mice are presented in Table 1; of note, data for males and females were pooled since no sex-associated differences were observed in SHAM and TAC mice of both genotypes (Supplementary Figure S2). In accordance with the present results, we previously demonstrated that there was no difference in the severity of AVS between younger male and female KD mice (Labbé et al., 2022).

In WT mice, TAC surgery significantly decreased LVFS (*p* < 0.05; Figure 4B; Table 1), LVEF (*p* < 0.05; Figure 4B; Table 1), LV lateral (*p* < 0.001) and septal (*p* < 0.01; Figure 4B; Table 1) wall contractility. In contrast, TAC did not worsen cardiac dysfunction already present in KD mice (Figure 4B; Table 1), except for a decreased stroke volume and an increased E deceleration rate in KD-TAC mice (Table 1). Thus, TAC surgery in WT mice globally mimics LV dysfunction observed in KD-SHAM mice (Figure 4B).

Gene expression of cardiac *Nox4* was then evaluated in both WT and KD mice subjected or not (SHAM) to TAC. Cardiac expression of *Nox4* mRNA was significantly and strongly negatively correlated with LVFS in KD mice (r = −0.8637, *n* = 14, *p* = 0.0001; Figure 4D), but not in WT mice (r = −0.3736, *n* = 13, *p* = 0.2095; Figure 4D). Similarly, cardiac expression of *Nox4* mRNA was significantly and strongly negatively correlated with LVEF in KD mice (r = −0.8681, *n* = 14, *p* = 0.0001; Figure 4E), but not in WT mice (r = −0.3736, *n* = 13, *p* = 0.2095; Figure 4E). Altogether, these data suggest that cardiac NOX4 is associated to the LV dysfunction observed in KD mice only. Of note, TAC surgery increased cardiac *Nox4* expression in WT mice (*p* < 0.05, *n* = 6-7; Supplementary Figure S3), but not in KD mice (*p* = 0.8518, *n* = 6–8; Supplementary Figure S3) where *Nox4* expression is already high.

As TAC surgery did not worsen cardiac dysfunction of KD mice, we focused on the SHAM group of mice to target NOX4. The impact of the injection of AAV9-shNOX4 on heart structure and LV systolic and diastolic function of KD mice and their age-matched WT littermates subjected to SHAM surgery are presented in Table 2. Cardiac *Nox4* expression was significantly reduced by a single injection of AAV9-shNOX4 in both WT (−33%, *n* = 6-7; *p* < 0.05; Figure 4F) and KD mice (−26%, *n* = 6; *p* < 0.05; Figure 4F). In contrast, the expression of *Nox4* in organs others than the heart (kidney and liver) was not notably affected by the shNOX4 (Supplementary Figure S4). Remarkably, whereas the shNOX4 had no effect on LV systolic function in WT-SHAM mice, except for a lower cardiac output (Table 2), targeting NOX4 fully reversed LV systolic dysfunction in KD-SHAM mice. Indeed, AAV9-shNOX4 significantly improved LVFS (+35%, *n* = 6-7, *p* < 0.01; Figure 4G; Table 2) and LVEF (+23%, *n* = 6-7, *p* < 0.01; Figure 4G; Table 2) in KD-SHAM mice; AAV9-shNOX4 also improved both LV lateral (+34%, *n* = 6-7, *p* < 0.01; Figure 4G; Table 2) and septal (+35%, *n* = 6-7, *p* < 0.01; Figure 4G; Table 2) wall contractility in KD-SHAM mice, normalizing values to those measured in WT mice. In contrast, LV diastolic function and LV structure were not affected by the shNOX4, neither in KD-SHAM nor in WT-SHAM mice (Table 2).

It is important to highlight that while the shNOX4 fully normalized LV systolic dysfunction in KD-SHAM mice, it did not correct AVS in these mice (Supplementary Table S3). In addition, the shNOX4 had no effect on LV systolic function in WT-TAC and KD-TAC mice (Supplementary Table S4). Thus, these data strongly suggest that cardiac NOX4 contributes, at least partly, to the LV systolic dysfunction observed in *Angptl2*-KD mice but does not contribute to the cardiac dysfunction induced by 6-week exposure to TAC.

### 3.3 The alleviation of systolic LV dysfunction observed in *Angptl2*-KD mice injected with the shNOX4 is associated with increased antioxidant markers

**FIGURE 5:**
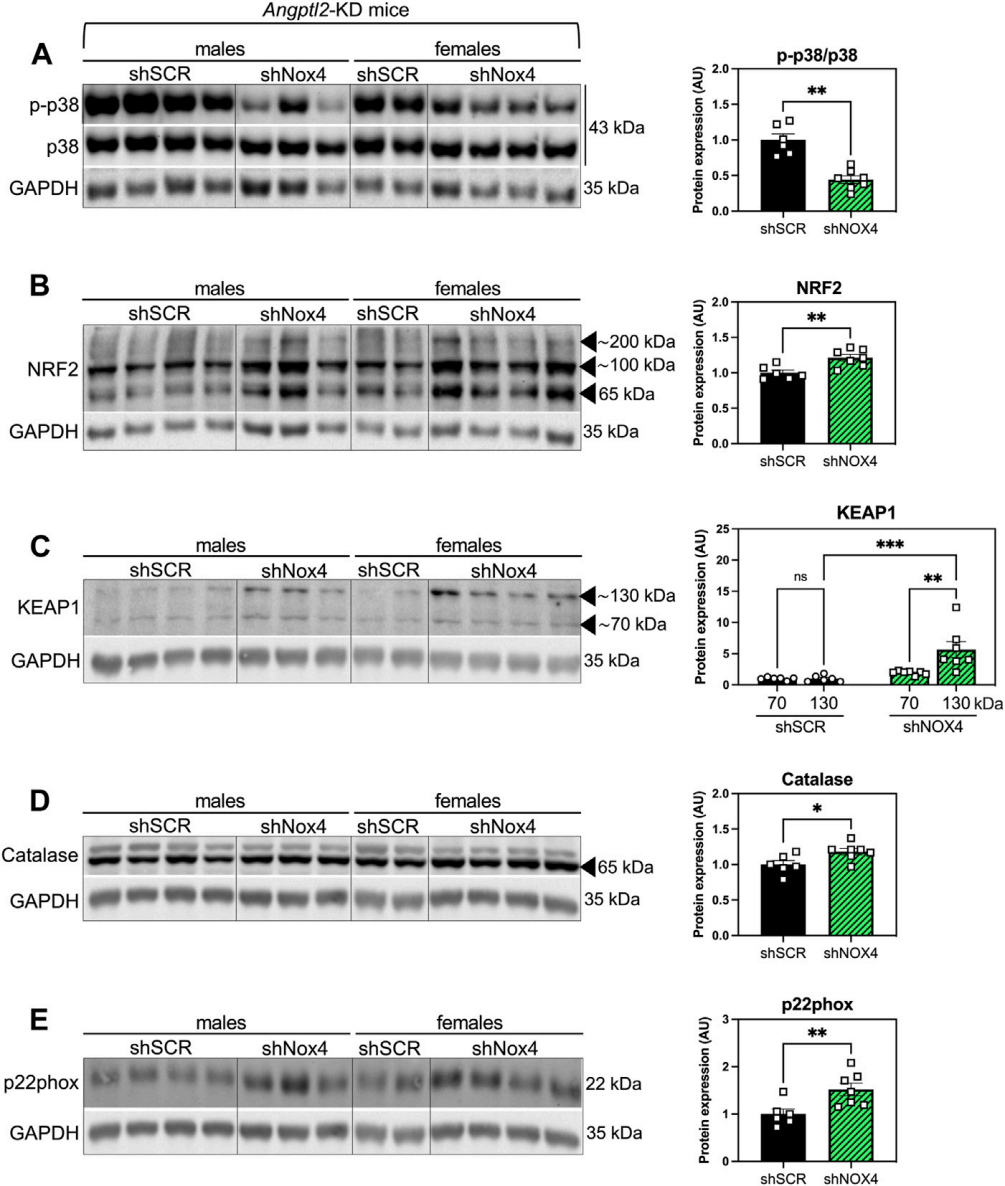
The alleviation of systolic LV dysfunction observed in *Angptl2*-KD mice injected with the shNOX4 is associated with increased antioxidant markers. Protein expression of **(A)** phospho (Thr180/Tyr182)-p38 (p-p38) and total p38, **(B)** NRF2, **(C)** KEAP1, **(D)** catalase and **(E)** p22phox, determined by Western Blot in hearts from KD mice subjected to SHAM surgery and injected with AAV9-shSCR or AAV9-shNOX4. GAPDH was used as a loading control, and the same image was used for **(D,E)** for illustrative purposes. Data are mean ± SEM of *n* = 6 KD mice (4 males and 2 females) in the AAV9-shSCR group, and *n* = 7 KD mice (3 males and 4 females) in the AAV9-shNOX4 group. *: *p* < 0.05 KD-shSCR vs. KD-shNOX4 mice; determined with Mann-Whitney U test.

To take a step further in the elucidation of the molecular mechanism underlying the alleviation of systolic dysfunction in KD mice injected with the shNOX4, we evaluated the levels of several proteins involved in oxidative stress response, including p38 signaling, NRF2/KEAP1, catalase and p22phox, in hearts of KD-SHAM mice treated with the shNOX4 compared to hearts of KD mice treated with the shSCR (Figure 5). The p38 signaling has been widely described as being involved in the cellular response to stress at many levels, including oxidative stress, and its activation is often correlated with heart diseases (Canovas and Nebreda, 2021). AAV9-shNOX4 strongly decreased the expression of phospho-p38 (p-p38/total p38) (−56%, *n* = 6-7, *p* < 0.01; Figure 5A), suggesting an alleviation of oxidative stress. Accordingly, cardiac expression of NRF2—the master regulator of cellular oxidative stress response (Ngo and Duennwald, 2022)—increased in KD-SHAM mice treated with the shNOX4 compared to those injected with the shSCR (+21%, *n* = 6-7, *p* < 0.01; Figure 5B). In addition, KEAP1, which regulates the activity of NRF2, was also highly increased, particularly its form at a molecular weight of ∼130/140 kDa (+466%, *n* = 6-7; *p* < 0.001; Figure 5C), *i.e.*, as a stable dimer that ubiquitinylates NRF2 to induce its degradation by the proteasome (Ogura et al., 2010). This rise in KEAP1 dimers suggests that the acute antioxidant response induced by targeting NOX4 promotes negative feedback inhibiting NRF2, allowing a progressive return to normal homeostasis. The expression of catalase, a crucial antioxidant enzyme that mitigates oxidative stress by degrading cellular hydrogen peroxide (Nandi et al., 2019), also increased in KD-SHAM mice injected with the shNOX4 (+18%, *n* = 6-7, *p* < 0.05; Figure 5D). Finally, AAV9-shNOX4 increased the expression of p22phox, one of the regulatory proteins for NOX1-4 (+52%, *n* = 6-7, *p* < 0.01; Figure 5E). Altogether, the increased expression of NRF2 and catalase–both markers of antioxidant response, and that of KEAP1 and p22phox–possibly reflecting a normalization feedback mechanism, suggest an alleviation of the oxidative stress, globally illustrated by the decrease of p38 signaling, in hearts of KD-SHAM mice injected with AAV9-shNOX4. This antioxidant response is concordant with a better systolic function in KD-SHAM mice injected with the shNOX4.

## 4 Discussion

**FIGURE 6:**
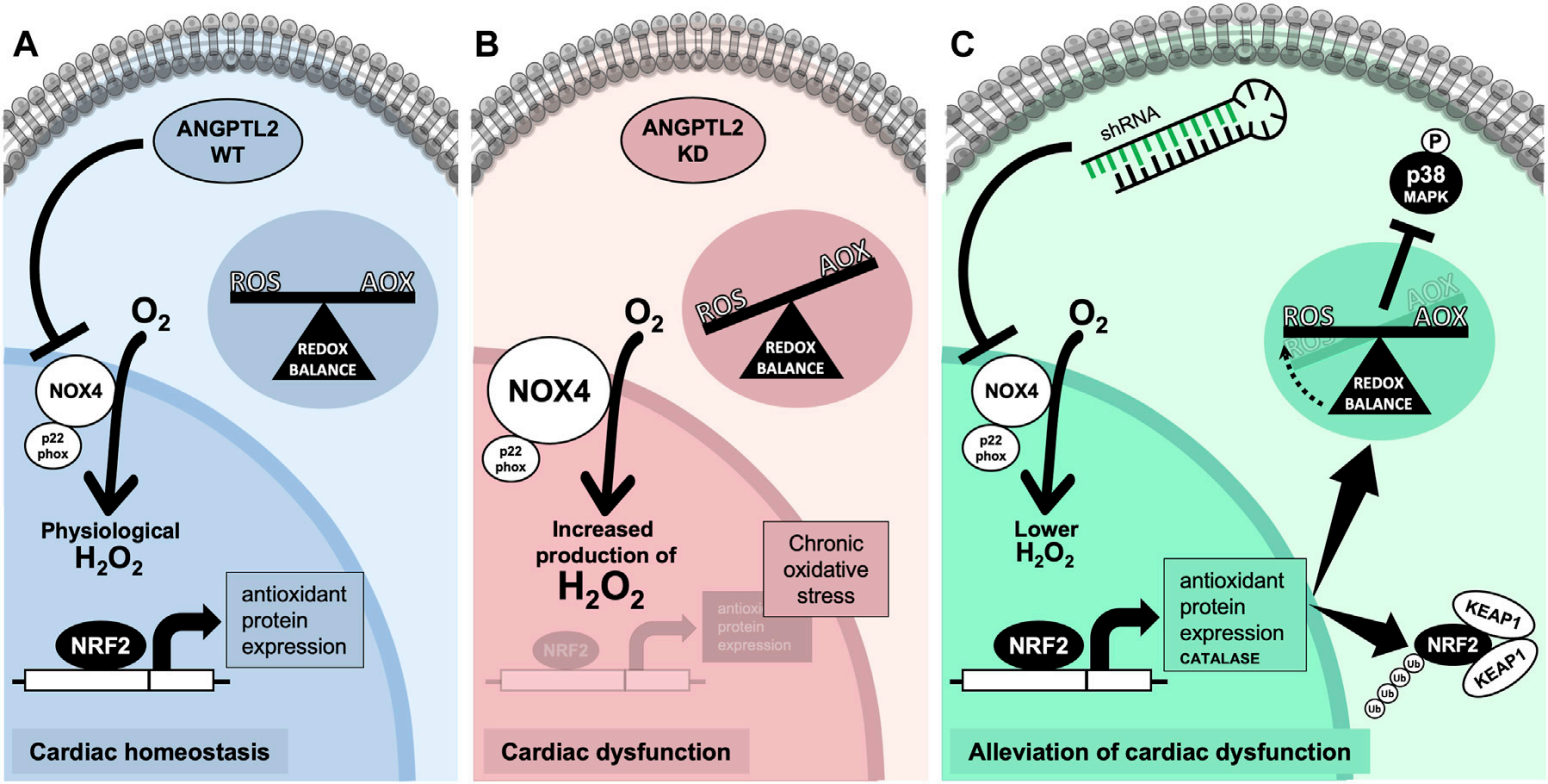
Proposed mechanism for ANGPTL2-mediated regulation of NOX4 activity. **(A)** In normal conditions, the presence of ANGPTL2 could allow negative feedback to regulate activated NOX4; this would prevent the overproduction of H_2_O_2_, a deleterious hypertrophic factor in cardiomyocytes. **(B)** In the absence of ANGPTL2, such as in KD mice, NOX4 activity would be increased, promoting high ROS levels which could progressively overwhelm antioxidant systems leading to an imbalance in favor of oxidative stress causing cellular damages, thus leading to LV systolic dysfunction in the long-term. **(C)** Decreasing cardiac *Nox4* expression with AAV9-shRNA may boost the antioxidant response, characterized by increased expression of the NRF2-KEAP1 pathway and catalase, and decreased p38 signaling, thus reversing the systolic dysfunction in KD mice. Parts of the figure were drawn by using pictures from Servier Medical Art. Servier Medical Art by Servier is licensed under a Creative Commons Attribution 3.0 Unported License.

The role of ANGPTL2 in cardiac function is relatively unknown. In patients, high circulating ANGPTL2 levels predict the occurrence of CVD (Hata et al., 2016) and could be a marker of heart failure (Huang et al., 2015). In adult mice, ANGPTL2 was shown to promote cardiac dysfunction and remodeling (Tian et al., 2016). Therefore, ANGPTL2 could be deleterious to the cardiac function and consequently, the absence of ANGPTL2 should be cardio-protective in adults. On the other hand, we recently reported that KD of *Angptl2* promoted an unexpected embryonic alteration in cardiac development leading to congenital AVS (Labbé et al., 2022). Accordingly, in the present study, adult KD mice developed an AVS, a small cardiac hypertrophy and a mild LV systolic dysfunction. We found that the upregulation of cardiac NOX4 expression plays a role in the cardiac dysfunction measured in *Angptl2*-KD mice. This is supported by our findings that i) cardiac NOX4 expression and activity are increased in KD mice, ii) ANGPTL2 and NOX4 proteins interact *in vitro*, iii) cardiac expression of *Nox4* correlates with the severity of LV dysfunction, and iv) cardiac repression of *Nox4* with AAV9-shNOX4 fully reversed LV dysfunction in *Angptl2*-KD mice. In other words, the presence of ANGPTL2 in cardiac cells may be beneficial by repressing the expression and activity of NOX4. We propose that in WT mice, the physiological role of cardiac ANGPTL2 is to directly downregulate NOX4 activity and contribute to preserve cardiac function (Figure 6A). In contrast, in KD mice, the systemic absence of ANGPTL2 could augment cardiac NOX4 activity and increase the release of hypertrophic H_2_O_2_; this could lead to the activation of a compensatory antioxidant response, which may be overwhelmed by the chronic oxidative stress associated with NOX4 free cycling, and progressively, lead to the development of a LV dysfunction in older mice (Figure 6B). Injection of AAV9-shNOX4 in KD mice could acutely boost antioxidant defenses by decreasing oxidative stress and, thus, improve systolic dysfunction (Figure 6C).

### 4.1 Cardiac NOX4 expression is increased in KD mice

How did *Angptl2*-KD promote cardiac dysfunction in mice? Accumulating evidence attest the importance of NOX-dependent oxidative stress—in particular from NOX2 and NOX4 that are highly expressed in the heart (Lassegue et al., 2012)—and ROS signaling in the pathogenesis of cardiac hypertrophy and failure (Chen et al., 2012). A fine balance between ROS and endogenous antioxidants is believed to exist, and any disturbance in this equilibrium in favor of ROS overflow may cause oxidative stress and damages prodromal to cardiac diseases (Singal et al., 1998). Considering the potential role of ANGPTL2 on NOX signaling that we previously described in the vascular endothelium of KD mice (Yu et al., 2015), and the contribution of NOXs in cardiac hypertrophy, fibrosis and progression towards heart failure [for review, (Bedard and Krause, 2007)], we investigated NOX expression in hearts from WT and KD mice. In line with our previous report of a higher vascular expression of NOX4 in KD mice (Yu et al., 2015), cardiac NOX4 expression was also increased in KD compared to WT mice. In contrast, cardiac NOX2 expression was not different between the two strains (Supplementary Figure S5), also in agreement with our previous report in arteries from KD mice (Yu et al., 2015). Hence, the lack of *Angptl2* favors *Nox4* overexpression; ANGPTL2 may bind NOX4 and repress and/or compartmentalize its expression as suggested by co-expression of ANGPTL2 and NOX4 in cardiac cells and by our data in HEK293 cells.

However, NOX4 involvement in cardiac remodeling/function is still controversial (Altenhofer et al., 2012; Chen et al., 2012; Touyz and Montezano, 2012; Varga et al., 2017). On the one hand, increased NOX4 expression and activity were reported in heart failure patients and in experimental models of cardiac hypertrophy (Dworakowski et al., 2008; Ago et al., 2010; Kuroda et al., 2010; Kuroda and Sadoshima, 2010; Matsushima et al., 2013; Frazziano et al., 2014; Matsushima et al., 2014; Zhao et al., 2015; Chen et al., 2023). On the other hand, cardiac function in transgenic *Nox4* mice was protected against abdominal and/or aortic banding, while cardiac specific and total KO of *Nox4* prevented angiogenesis, antioxidant responses and led to worse cardiac remodeling (Zhang et al., 2010; Zhang et al., 2013; Zhang et al., 2018). In the present study, cardiac specific decrease of *Nox4* expression by AAV9-shRNA abolished the mild dysfunctional cardiac phenotype of KD mice. Thus, our data support a (mild) deleterious impact of NOX4 in the heart in the absence of ANGPTL2.

### 4.2 The interplay between cardiac ANGPTL2, NOX4 and ROS regulation

It is well established that mitochondria are a critical player in the redox regulation in the myocardium (Peoples et al., 2019). Here, we demonstrated an increased expression and activity of NOX4 in hearts of KD mice; we did not investigate the specific localization of NOX4, that can be expressed in different cellular organelles such as the mitochondria, the endoplasmic reticulum or the nucleus. In the specific context of myocardium injury/reperfusion (I/R), the regulatory mechanism and the function of NOX4 in various subcellular localizations and organelles are still unclear (Matsushima and Sadoshima, 2022). Mitochondrial-NOX4 could potentially exert a detrimental effect during myocardial I/R by causing ROS-induced mitochondrial dysfunction; in contrast, endoplasmic reticulum-NOX4 could be potentially protective against I/R through regulation of autophagy (Matsushima and Sadoshima, 2022). Based on this data, it is plausible that the effect of KD of ANGPTL2 could differ according to the subcellular localizations of NOX4, but this remains to be demonstrated.

In the present study, KD of ANGPTL2 was associated with an increased NOX4 expression and activity, suggesting that the presence of ANGPTL2 in cardiac cells may be beneficial by repressing the expression and activity of NOX4, thus maintaining physiological levels of ROS. However, the specific involvement of ANGPTL2 in the regulation of oxidative stress is ill defined. Indeed, whereas ANGPTL2 is well known to promote inflammation, only a few data reported its pro-oxidative effect. In skin tissues from K14-*Angptl2* transgenic mice, Aoi et al. showed for the first time a higher level of lipid peroxidation that was sensitive to an antioxidant treatment (Aoi et al., 2014). This pro-oxidative property of ANGPTL2 likely synergizes with its pro-inflammatory effect to promote carcinogenesis and metastasis (Aoi et al., 2011). Similarly, ANGPTL2 suppression in skeletal myocytes led to lower inflammation and ROS levels (Zhao et al., 2018). In addition, whether ANGPTL2-induced oxidative stress contributes to cardiovascular diseases and atherogenesis remains to be demonstrated. It could be speculated that ANGPTL2-induced oxidative stress is at least partially responsible for endothelial dysfunction since it could be reversed by an antioxidant (Yu et al., 2014). Furthermore, we reported that ANGPTL2 expression was increased in a pro-oxidative environment such as smoking (Farhat et al., 2008), suggesting a relationship between ANGPTL2 and oxidative stress.

Whether ANGPTL2-induced ROS originate from mitochondria is unknown. To the best of our knowledge, there are no reports on the relationship between ANGPTL2 and mitochondrial ROS production. We previously reported that *Angptl2*-knockdown protects cerebral endothelial function via an apocynin- and PEG-catalase-sensitive dilatory pathway, suggesting that *Angptl2*-KD protects endothelial function through an alternative NOX4 dilatory pathway (Yu et al., 2015). Of note, we observed that this protective pathway was insensitive to MitoTEMPO, a scavenger of mitochondrial ROS, suggesting a source of ROS independent of the mitrochondria. Tian et al. (Tian et al., 2016) showed that ANGPTL2 suppression in cardiomyocytes increases expression of genes promoting β-fatty acid oxidation, mitochondrial biogenesis and intracellular ATP production. Inversely, ANGPTL2 overexpression lowered expression of genes promoting β-fatty acid oxidation, reduced mitochondrial biogenesis and energy metabolism. In this latter study, however, oxidative stress was not examined.

In summary, despite the facts that ROS originating from the mitochondria is an important mechanism of disease in the cardiovascular system, and that NOX4 is mostly localized in mitochondria, it remains to determine where and how NOX4 and ANGPTL2 interact in cardiac cells to regulate ROS production, cardiac remodeling and function.

### 4.3 Targeting cardiac NOX4 reversed LV systolic dysfunction in *Angptl2*-KD mice

In our study, targeting NOX4 with the shRNA only partially reduced NOX4 levels, normalizing them to WT levels. The KO mouse studied by Kuroda et al. (Kuroda et al., 2010) (supporting that NOX4 is deleterious to the heart) was generated by targeting exon 9, a method that could potentially result in a truncated protein with some biological effects. In contrast, the *Nox4*-Null mouse from Zhang et al. (Zhang et al., 2018) (supporting that NOX4 is cardioprotective) was generated by targeting the translation initiation site and the first two exons of the gene, leading to a complete absence of NOX4. Thus, distinct genetic engineering of mice models—the total absence, or residual expression levels of *Nox4*—could therefore explain conflicting results in the literature concerning the cardiac role of NOX4. In addition, at least 5 splice variants for NOX4 have been reported in human and rodent hearts (Varga et al., 2017), of which certain lack FADH and NAD(P)H binding sites and/or transmembrane domains; these variants possibly exert different biological effects in distinct cellular compartments, and have been associated with either increased or decreased ROS levels *in vitro* (Goyal et al., 2005). The latter could easily explain the differences in *Nox4*-Null mice compared to less drastic models with residual biologically active truncated proteins, even with the subtlest expression levels. NOX4, in opposition to other NOXs, has the particularity to be constitutively active, and its activity is strictly determined by mRNA levels, with a close temporal correlation between induction of *Nox4* mRNA and H_2_O_2_ production (Serrander et al., 2007).

Another explanation for the discrepancies observed in the beneficial and deleterious effects of NOX4 in mice subjected to TAC surgery could be the severity and the timing of the procedure (Zhang et al., 2018). In our study, 6 weeks of partial ligation of the transverse aorta successfully generated a pressure overload prompting cardiac dysfunction in WT mice; in *Angptl2*-KD mice, however, this dysfunction was not worsened despite the application of a similar hemodynamic stress to both WT and KD mice (Supplementary Table S2). LV hypertrophy and cardiac remodeling observed in WT-TAC mice mimicked the mild cardiac dysfunction of adult KD mice. In comparison, Kuroda et al. used a very severe TAC model resulting in >40% decrease in LVEF (Kuroda et al., 2010), whereas that used by Zhang et al., as in our study, is more moderate, resulting in 20% and ∼15% decrease in LVEF, respectively (Zhang et al., 2018). Of note, in our study, TAC increased cardiac Nox4 expression levels in WT, but not further in KD mice (Supplementary Figure S3). Paradoxically, AAV9-shNOX4 did not affect the cardiac function of WT mice, neither under SHAM nor TAC surgery, suggesting that NOX4 does not contribute to the cardiac dysfunction after 6-week of pressure overload induced by TAC. Clearly, reduction of NOX4 expression in the context of TAC-associated pressure overload has no consequence on cardiac function. However, comparing our results with previous studies is difficult, as most of them explored the impact of pressure overload in a global or cardiomyocyte-specific deletion of *Nox4* in engineered mice, with total—or almost total—abolition of *Nox4* expression, and with a wide range of mild to severe stimuli. Here, we report a more subtle, short-term inhibition of *Nox4* expression, using adeno-associated viruses sufficient to reverse the ∼30% increase of NOX4 expression observed in KD mice compared to WT, thus resulting in the correction of the mild LV systolic dysfunction, with echo parameters returning to levels observed in WT mice.

At baseline, *i.e.*, in the absence of pressure overload, Zhang et al. observed that their *Nox4*-transgenic mice—with significant elevation of myocardial H_2_O_2_ production—had a slight cardiac hypertrophy in 12-month old mice compared with WT littermates, although *Nox4*-transgenic mice were grossly normal and showed no cardiac dysfunction at 3 months (Zhang et al., 2010), suggesting a deleterious chronic cardiac effect of NOX4. Similarly, Ago et al. reported that, although not different from the non-transgenic mice at a “young” age (unspecified), their 13-14 months of age transgenic mice with cardiac-specific overexpression of *Nox4*—1.3 to 2.1-fold increase in NOX4 protein expression, comparable to the ∼1.4-fold increase in hearts of our KD mice–exhibit a decrease in ejection fraction, with an increase in myocytes area and apoptosis, fibrosis and oxidative stress (Ago et al., 2010). In non-transgenic mice, cardiac NOX4 expression is also increased with age (Ago et al., 2010). Together, these results suggest that long-term overexpression of NOX4 could be deleterious for cardiac function, in accordance with the cardiac phenotype observed in 7-month-old *Angptl2*-KD mice.

### 4.4 Reduction of NOX4 promoted an antioxidant response

Mechanistically, our data suggest that AAV9-shNOX4 abolished the dysfunctional cardiac phenotype of KD mice by revealing an antioxidant response characterized by an increased expression of the NRF2-KEAP1 pathway and catalase. NRF2 is the master regulator of cellular oxidative stress response (Ngo and Duennwald, 2022) and KEAP1 regulates the activity of NRF2. Yet, the increased expression of these antioxidant markers in the hearts of KD mice injected with the shNOX4 is counterintuitive at first. Indeed, previous work demonstrated that endogenous NOX4 is required to promote NRF2 activation during pressure overload in hearts *in vivo* (Smyrnias et al., 2015). This suggests that after an acute stress, endogenous NOX4 activates NRF2. In hearts of KD mice, however, chronic H_2_O_2_ generation associated with the overexpression of NOX4 likely overwhelmed the antioxidant response capacity, preventing NRF2 signaling. On the other hand, reduction in *Nox4* expression in KD mice promoted NRF2 expression. Interestingly, reduction in *Nox4* expression in KD mice also induced the rise in KEAP1 dimers, suggesting that the acute antioxidant response induced by targeting NOX4 promotes negative feedback inhibiting NRF2, allowing a progressive return to homeostasis. The alleviation of the oxidative stress in hearts of these KD mice is globally illustrated by the strong decrease in cardiac p38 signaling. ROS have been reported to activate p38 in various pathological contexts, and the activation of this pathway often correlates with cardiac pathologies, supporting the efforts to use p38 inhibitors in clinic (Canovas and Nebreda, 2021). Accordingly, a decrease in p38 signaling—using pharmacological inhibitors or genetic methods—was associated with an improved cardiac function in several animal models of myocardial infarction and heart failure (Ma et al., 1999; Liu et al., 2005). Altogether, these data suggest that the absence of ANGPTL2 in cardiac cells may be deleterious to LV function by up-regulating NOX4 expression and activity, leading to oxidative stress and cardiac dysfunction. Accordingly, reducing cardiac NOX4 expression in KD mice promoted an antioxidant response and reversed LV systolic dysfunction.

### 4.5 Limitations of the study

First, how ANGPTL2 mechanistically regulates NOX4 expression and/or activity remains to be elucidated. NOX4 and ANGPTL2 could directly interact into the cytosol, as NOX4 can be expressed in cellular organelles such as the mitochondria, the endoplasmic reticulum or the nucleus (Bedard and Krause, 2007). In the present study, whether NOX4 was sub-localized in mitochondria or in endoplasmic reticulum remains to be elucidated. Second, although the cardiac phenotype observed in *Angptl2*-KD mice is clearly the combined outcome of knock-down of ANGPTL2 in all cardiac cells, including endothelial cells, vascular smooth muscle cells, fibroblasts and cardiomyocytes, the role played by each cellular subtype in LV dysfunction was beyond the scope of this study. Third, even if we observed an interaction between NOX4 and ANGPTL2 in HEK293 cells in the co-IP assay, our experimental conditions did not permit to decipher whether ANGPTL2 and NOX4 interact in the cytosol or at the level of membranes (cytosolic or organelle membranes). In addition, the binding of NOX4 and ANGPTL2 in transfected HEK293 cells does not fully reflect what may occur in cardiac cells. Fourth, the limited number of male and female mice used in the present study prevented us from observing any sex-related differences that may occur in ANGPTL2-mediated NOX4 regulation; it would be important to assess a potential sexual dimorphism in these KD mice since sex-related differences have been reported for many redox-regulated molecules. Overall, whereas our work suggests that NOX4 is a key mediator of LV dysfunction in ANGPTL2-deficient mice, additional experiments to support ANGPTL2 negative regulation of NOX4 are needed.

### 4.6 Conclusion

We demonstrated that adult *Angptl2*-KD mice exhibit LV cardiac dysfunction that is likely triggered by the overactivity of NOX4: cardiac *Nox4* expression was correlated to the severity of LV cardiac dysfunction, whereas cardiac specific reduction of *Nox4* reversed the deleterious effects of *Angptl2* knockdown on cardiac dysfunction. Based on multiple evidence of an association between ANGPTL2 and the prevalence and severity of CVD, decreasing ANGPTL2 levels has been proposed as a potential therapeutic strategy for the treatment and prevention of CVD. Our present data highlight that, in mice, the chronic genetic deletion of ANGPTL2 generates a mild cardiac dysfunction, suggesting that the presence of ANGPTL2 in cardiac cells may be beneficial by repressing the expression and activity of NOX4. Whether therapeutic targeting of cardiac ANGPTL2 is safe therefore deserves further investigation.

## Data Availability

The original contributions presented in the study are included in the article/Supplementary Material, further inquiries can be directed to the corresponding author.
