# Supplementary material for: Knockdown of ANGPTL2 promotes left ventricular systolic dysfunction by upregulation of NOX4 in mice
Source: Front Physiol. 2024 Feb 15;15:1320065. doi: 10.3389/fphys.2024.1320065 (PMC10902461; doi:10.3389/fphys.2024.1320065)
Supplement: Supplementary file 1 [file DataSheet1.PDF]

Supplementary Material

Table S1: Echocardiographic evaluation of aortic valve function in *Angptl2*-KD mice and their age-matched WT littermates at 7 months.

Table S2: Echocardiographic evaluation of aortic banding in *Angptl2*-KD mice and their age-matched WT littermates.

Table S3: Echocardiographic evaluation of aortic valve function in *Angptl2*-KD mice and their age-matched WT littermates after targeting cardiac NOX4 expression.

Table S4: Echocardiographic evaluation of cardiac function in *Angptl2*-KD mice and their age-matched WT littermates subjected to TAC surgery after targeting cardiac NOX4 expression.

Figure S1: KD mice exhibit a slight cardiac hypertrophy with a small LV remodelling.

Figure S2: No sex-related differences were observed in echocardiographic parameters between WT and KD mice.

Figure S3: Effect of the TAC surgery on cardiac gene expression of *Nox4* in WT and KD mice.

Figure S4: Effect of the AAV9-shNOX4 injection on the expression of *Nox4* in liver and kidney of WT and KD mice.

Figure S5: NOX2 gene and protein expression in hearts of WT and KD mice subjected to SHAM surgery.

Figure S6: Unedited blots/gels for Figure 1, Figure 3 and Figure 5.

Table S1: Echocardiographic evaluation of aortic valve function in *Angptl2*-KD mice and their age-matched WT littermates at 7 months.

	**WT-SHAM-shSCR** (6)	**KD-SHAM-shSCR** (6)	**WT-TAC-shSCR** (7)	**KD-TAC-shSCR** (8)
Aortic valve area (mm^2)	0.60±0.07	0.45±0.08	0.68±0.05	0.45±0.05 **$**
Peak velocity (cm/s)	94.46±5.30	169.9±33.15 **	72.38±4.02	107.9±7.36
Mean gradient (mm Hg)	2.16±0.23	8.38±2.86 **	1.27±0.16	2.86±0.39
Leaflet thickness (mm)	0.14±0.02	0.19±0.02	0.14±0.02	0.18±0.03

Data are mean±SEM of (n) WT and KD mice.
* : WT-SHAM *vs*. KD-SHAM (2-way ANOVA, Bonferroni post-hoc test)
$: WT-TAC *vs*. KD-TAC (2-way ANOVA, Bonferroni post-hoc test)

Table S2: Echocardiographic evaluation of aortic banding in *Angptl2*-KD mice and their age-matched WT littermates.

	**WT-SHAM-shSCR** (6)	**KD-SHAM-shSCR** (6)	**WT-TAC-shSCR** (7)	**KD-TAC-shSCR** (8)
Aortic diameter - Banding (mm)	1.22±0.08	1.13±0.15	0.67±0.07 ***	0.64±0.05 **$$** (7)
Peak velocity - Banding (cm/s)	67.30± 4.13	76.46±14.91	303.97±16.16 ****	252.00±25.91 **$$$$**
Peak gradient - Banding (mm Hg)	2.09±0.19	2.79±1.05	37.59±3.87 ****	27.28±4.95 **$$$**
Mean gradient - Banding (mm Hg)	1.33±0.28	1.41±0.51	22.67±3.04 ****	16.63±2.75 **$$$**

Data are mean±SEM of (n) WT and KD mice.
* : WT-SHAM *vs*. WT-TAC (2-way ANOVA, Bonferroni post-hoc test)
$: KD-SHAM *vs*. KD-TAC (2-way ANOVA, Bonferroni post-hoc test)

Table S3: Echocardiographic evaluation of aortic valve function in *Angptl2*-KD mice and their age-matched WT littermates after targeting cardiac NOX4 expression.

	**WT-SHAM-shSCR** (6)	**KD-SHAM-shSCR** (6)	**WT-SHAM-shNOX4** (8)	**KD-SHAM-shNOX4** (7)
Aortic valve area (mm^2)	0.60±0.06	0.45±0.08	0.62±0.04	0.36±0.10 *
Peak velocity (cm/s)	94.46±5.30	169.85±33.15	91.24±4.50	195.64±32.91 **
Mean gradient (mm Hg)	2.16±0.23	8.38±2.86	1.99±0.18	11.62±3.23 **
Leaflet thickness (mm)	0.14±0.02	0.19±0.02	0.15±0.01	0.20±0.03

Data are mean±SEM of (n) WT and KD mice.
* : WT-shNOX4 *vs*. KD-shNOX4 (2-way ANOVA, Bonferroni post-hoc test)

Table S4: Echocardiographic evaluation of cardiac function in *Angptl2*-KD mice and their age-matched WT littermates subjected to TAC surgery after targeting cardiac NOX4 expression.

		**WT-TAC-shSCR** (7)	**WT-TAC-shNOX4** (7)	**KD-TAC-shSCR** (8)	**KD-TAC-shNOX4** (8)
**LV structure**	*LV mass (mg)*	167.9±11.3	180.0±7.5	151.3±12.4	181.2±12.5
	LV mass/body weight (mg/g)	6.2±0.5	6.2±0.5	5.5±0.3	6.7±0.4 #
	LV anterior wall thickness (mm)	1.03±0.04	0.84±0.07	0.98±0.04	0.97±0.04
	LV posterior wall thickness (mm)	0.90±0.03	1.00±0.03	0.84±0.04	0.97±0.04 #
**LV systolic function**	*Fractional shortening (%)*	27.6±2.3	30.8±4.8	25.4±2.5	23.9±2.1
	Ejection fraction (%)	59.9±3.5	62.7±6.7	56.1±3.9	53.6±4.0
	Cardiac output (mL/min)	10.4±1.0	10.2±1.1	10.4±0.7	8.7±0.5
	Stroke volume (µl)	26.4±2.7	29.7±3.4	25.4±1.2	22.4±1.7
	Lateral wall contractility (cm/s)	1.76±0.12	1.88±0.14	1.58±0.12	1.41±0.11 **$**
	Septal wall contractility (cm/s)	1.93±0.15	2.12±0.18	2.10±0.20	1.75±0.13
	LV end-diastolic volume (µL)	193.0±23.8	190.0±19.5	181.1±13.9	212.4±11.5
	LV end-systolic volume (µL)	81.1±15.9	74.7±15.3	123.1±36.3	99.8±11.6
**LV diastolic function**	*E/A ratio*	1.2 (1)	3.6±1.0 (5)	3.6±1.9 (3)	2.5±0.3 (2)
	E/E' ratio	29.9 (1)	33.5±3.5 (5)	40.9±2.0 (4)	41.1±4.0 (4)
	E velocity (cm/s)	87.4±6.5	79.2±3.7	94.0±5.0 (7)	92.9±4.6 **$**
	A velocity (cm/s)	52.3 (1)	34.9±11.8 (5)	40.8±14.7 (3)	31.7±6.3 (2)
	E deceleration time (ms)	32.3±2.6	25.6±3.5	28.8±2.0 (7)	29.3±2.2
	E deceleration rate (m/s^2)	28.2±3.1	33.7±3.4	34.0±3.8 (7)	33.0±3.0

Data are mean±SEM of (n) WT and KD mice.
$: WT-TAC-shNOX4 *vs*. KD-TAC-shNOX4 (2-way ANOVA, Bonferroni post-hoc test)
# : KD-TAC-shSCR *vs*. KD-TAC-shNOX4 (2-way ANOVA, Bonferroni post-hoc test)

Figure S1

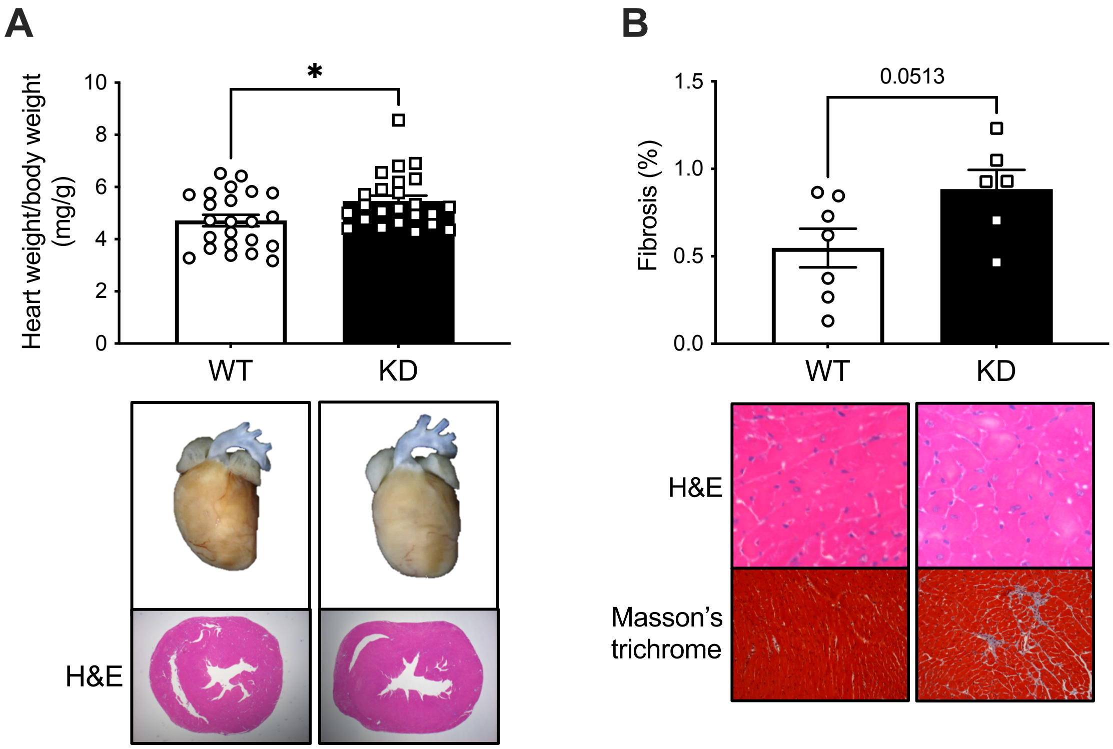

Figure S1: KD mice exhibit a slight cardiac hypertrophy with a small LV remodelling.

(**A**) Heart weight/body weight ratio in KD mice, and representative images of whole hearts and sections stained with Masson's trichrome (bottom panel); Data are mean±SEM of n=23 male WT mice and n=24 male KD mice. *: $p<0.05$ *vs.* WT mice determined with Mann-Whitney U test. (**B**) Quantification of fibrosis in heart from WT and KD mice, and representative images visualized by histology in cardiac sections stained with Hematoxylin and Eosin (H&E) or Masson's trichrome (bottom panel); Data are mean±SEM of n=7 male WT mice and n=6 male KD mice. $p=.0513$ *vs.* WT mice determined with Mann-Whitney U test.

Figure S2

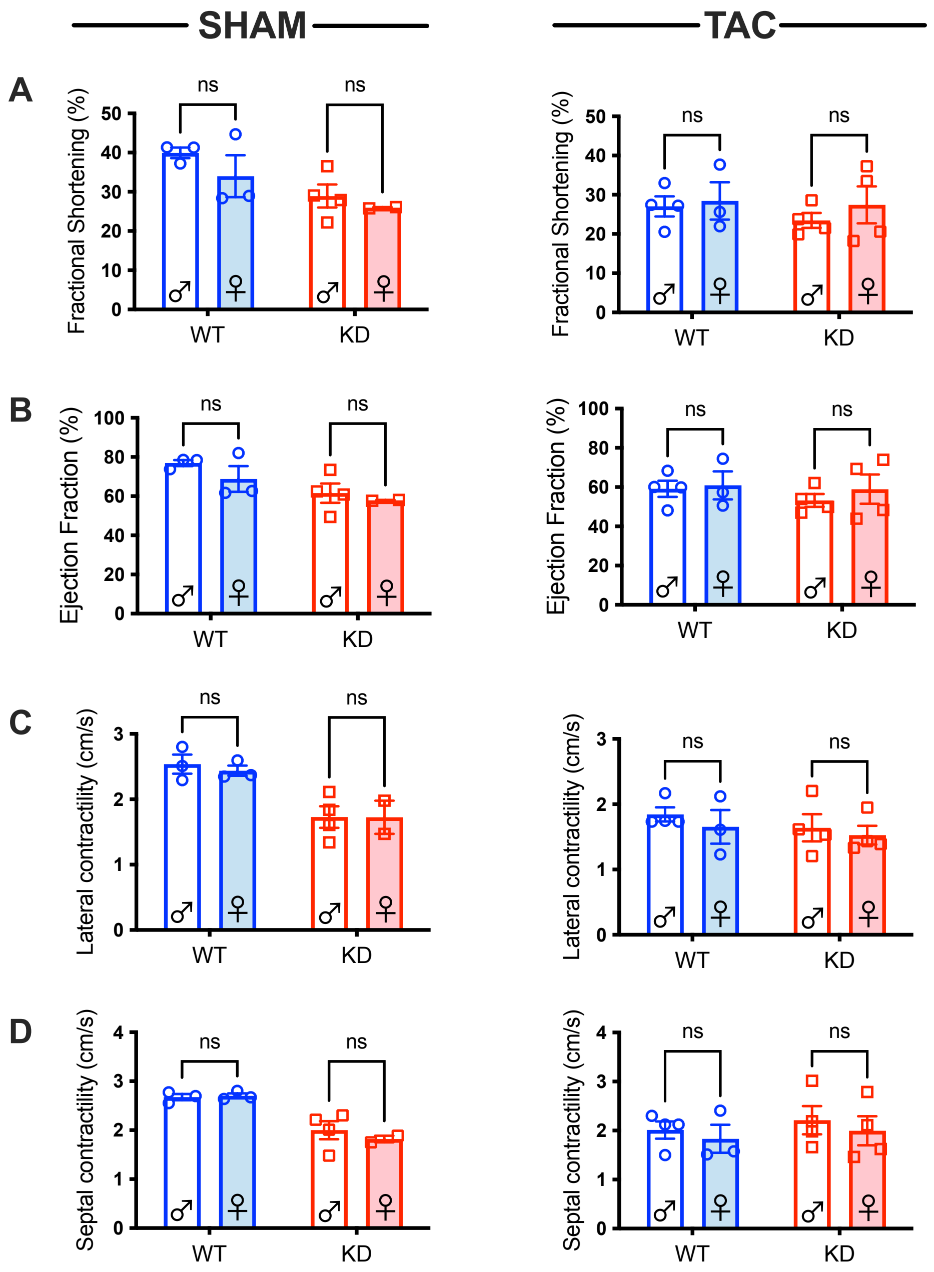

Figure S2: No sex-related differences were observed in echocardiographic parameters between WT and KD mice.

Echocardiographic measurements of **(A)** fractional shortening, **(B)** ejection fraction, **(C)** lateral and **(D)** septal contractility in male and female WT and KD mice subjected to SHAM or TAC surgery. For SHAM surgery: Data are mean±SEM of n=3 male WT mice; n=3 female WT mice; n=4 male KD mice and n=2 female KD mice; For TAC surgery: Data are mean±SEM of n=4 male WT mice; n=3 female WT mice; n=4 male KD mice; n=4 female KD mice. ns: non-significant, 2-way ANOVA, Bonferroni post-hoc test.

Figure S3

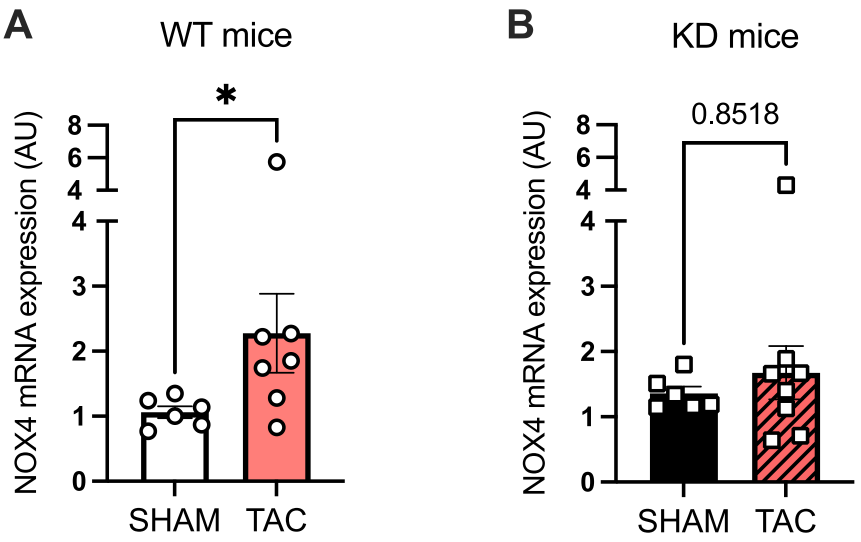

Figure S3: Effect of the TAC surgery on cardiac gene expression of *Nox4* in WT and KD mice.

Cardiac *Nox4* expression in **(A)** WT mice and **(B)** KD mice subjected to TAC or not (SHAM) surgery. Data are means±SEM of n=6-7 mice for WT and n=6-8 mice for KD.
*: p<0.05 determined with Mann-Whitney test.

Figure S4

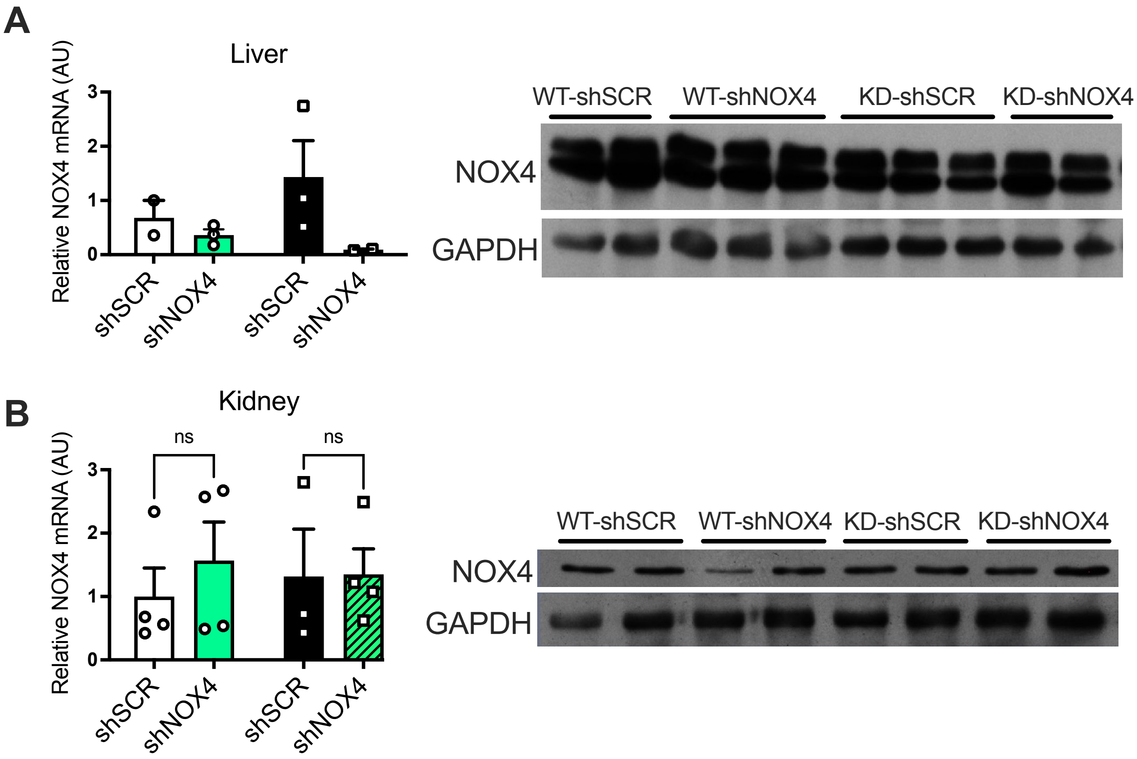

Figure S4: Effect of the AAV9-shNOX4 injection on the expression of *Nox4* in liver and kidney of WT and KD mice.

Gene and protein expression of NOX4 in **(A)** liver and **(B)** kidney of WT and KD mice injected with the AAV9-shSCR/NOX4. Data are means±SEM of n=2-4 mice for WT-shSCR; n=3-4 mice for WT-shNOX4; n=3 mice for KD-shSCR and n=2-4 mice for KD-shNOX4. ns: determined with 2-way ANOVA, Bonferroni post-hoc test.

Figure S5

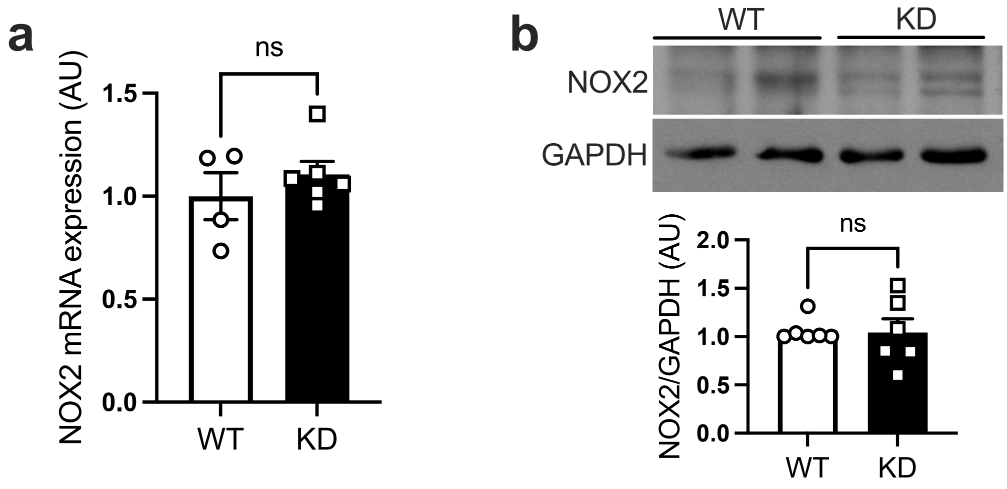

Figure S5: NOX2 gene and protein expression in hearts of WT and KD mice subjected to SHAM surgery.

Gene **(A)** and protein **(B)** expression of NOX2 in hearts of WT and KD mice subjected to SHAM surgery. Data are means±SEM of n=4-6 mice for WT and n=6 mice for KD.
ns: determined with Mann-Whitney test.

Figure S6

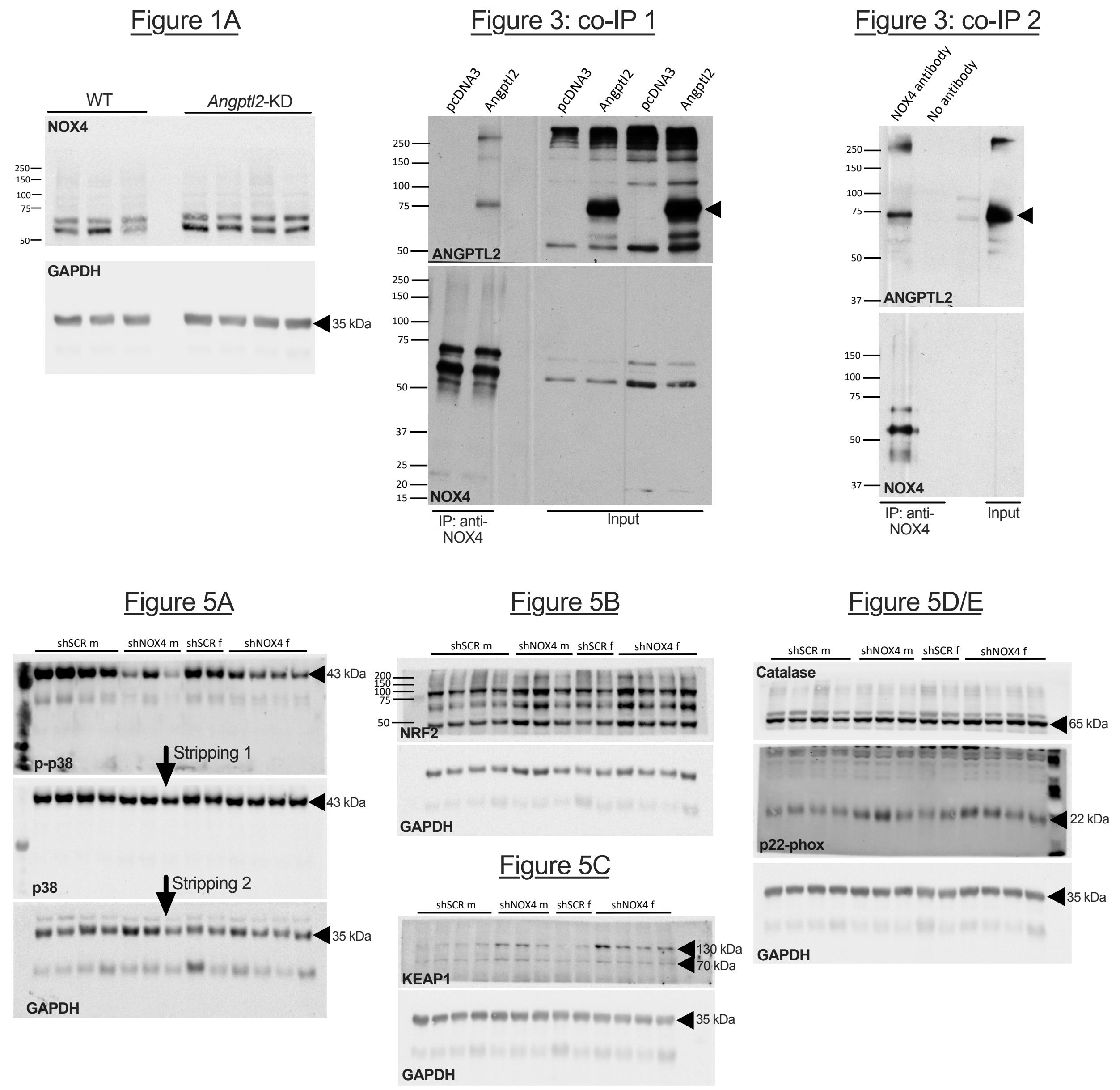

Figure S6: Unedited blots/gels for Figure 1, Figure 3 and Figure 5.
