# Supplementary figures and images for: Knockdown of ANGPTL2 promotes left ventricular systolic dysfunction by upregulation of NOX4 in mice
Source: Front Physiol. 2024 Feb 15;15:1320065. doi: 10.3389/fphys.2024.1320065 (PMC10902461; doi:10.3389/fphys.2024.1320065)

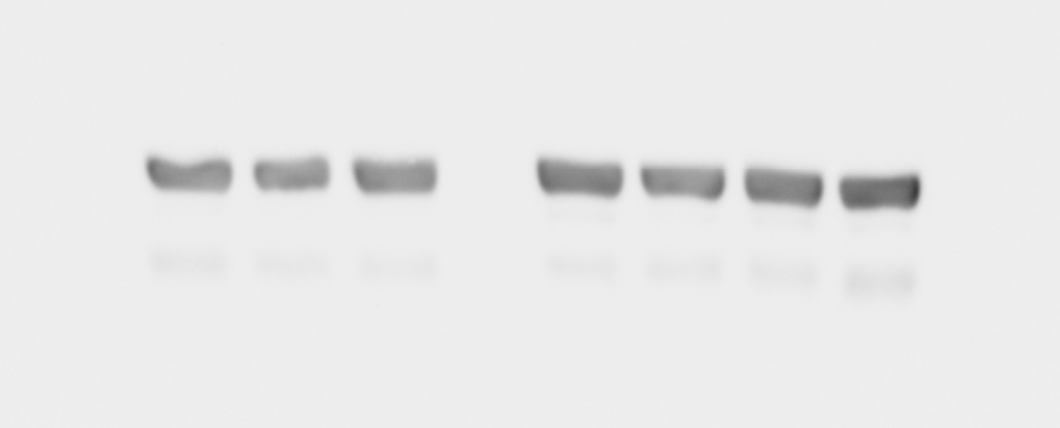

Supplement: Supplementary file 2 [file DataSheet2.ZIP › Original images for Western Blot/Figure 1/Figure 1 - GAPDH.tif]

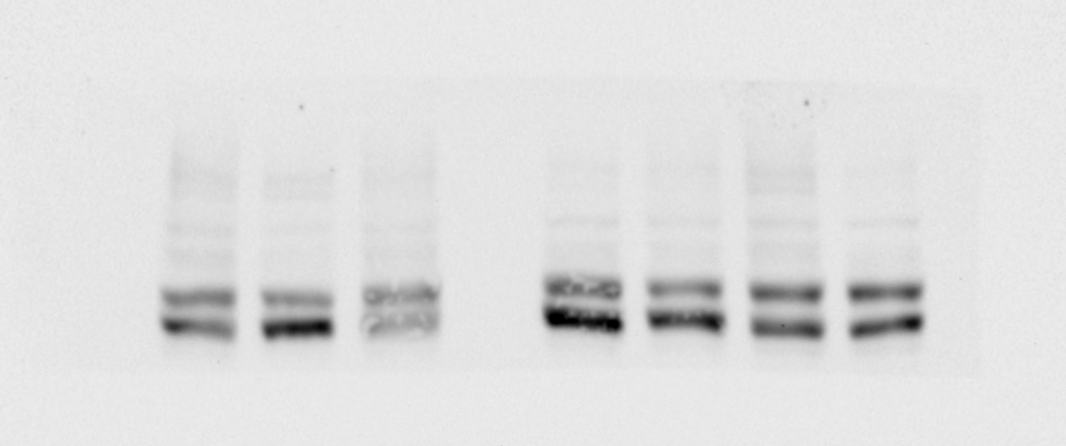

Supplement: Supplementary file 2 [file DataSheet2.ZIP › Original images for Western Blot/Figure 1/Figure 1 - NOX4.tif]

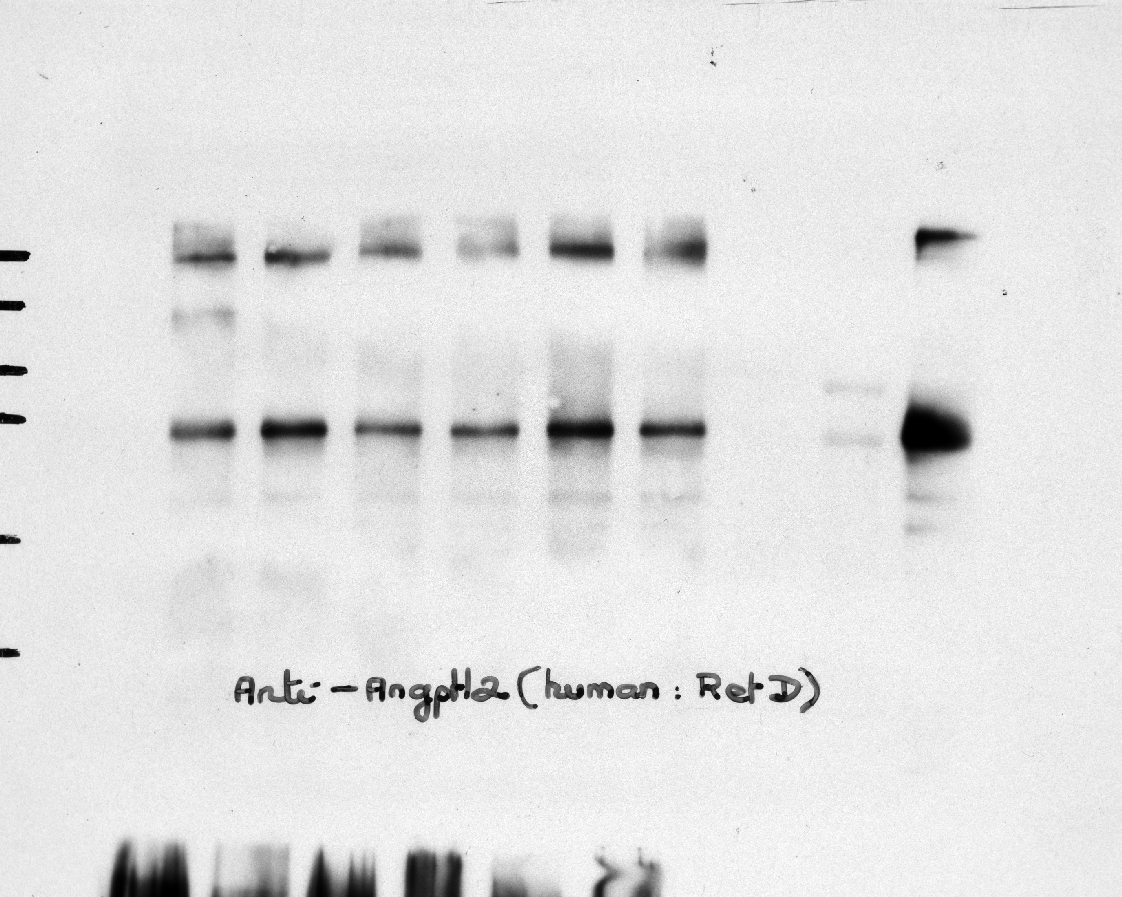

Supplement: Supplementary file 2 [file DataSheet2.ZIP › Original images for Western Blot/Figure 3/Figure 3 - ANGPTL2 bis.tif]

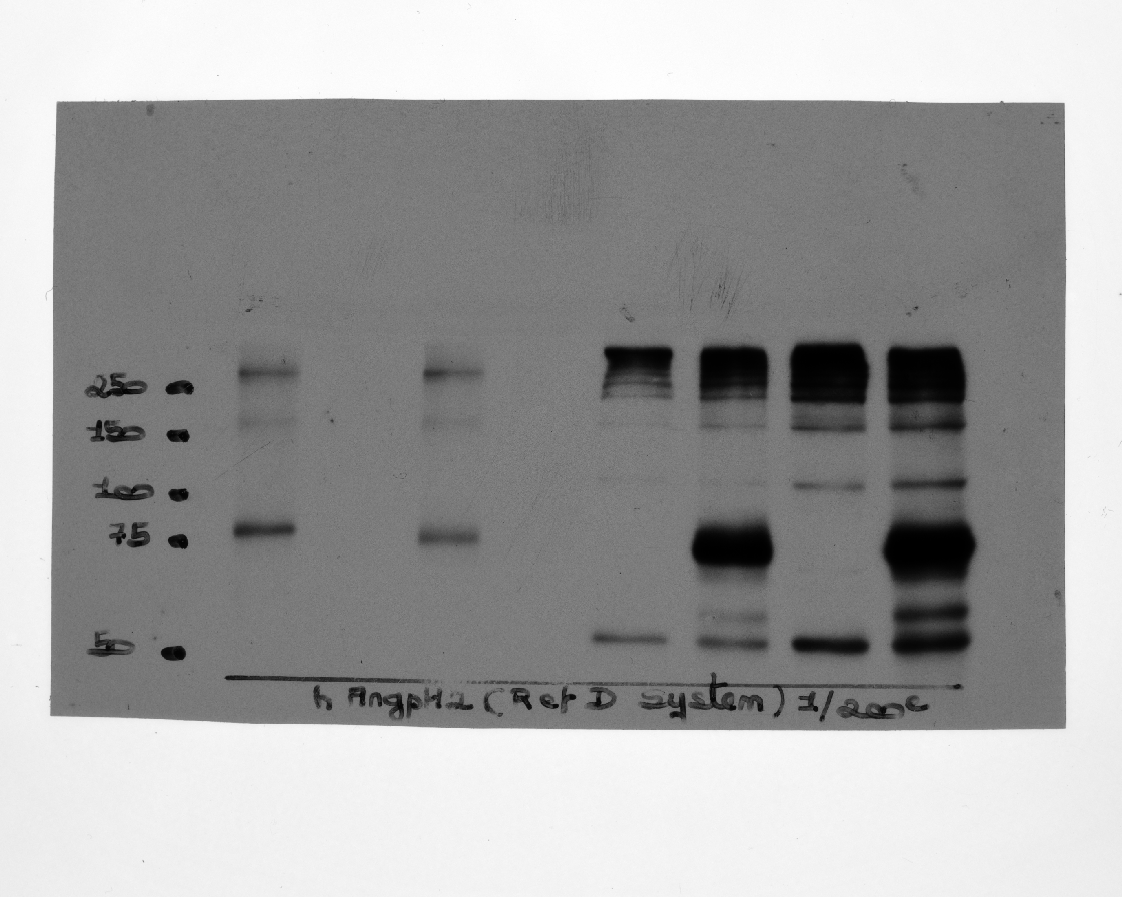

Supplement: Supplementary file 2 [file DataSheet2.ZIP › Original images for Western Blot/Figure 3/Figure 3 - ANGPTL2.tif]

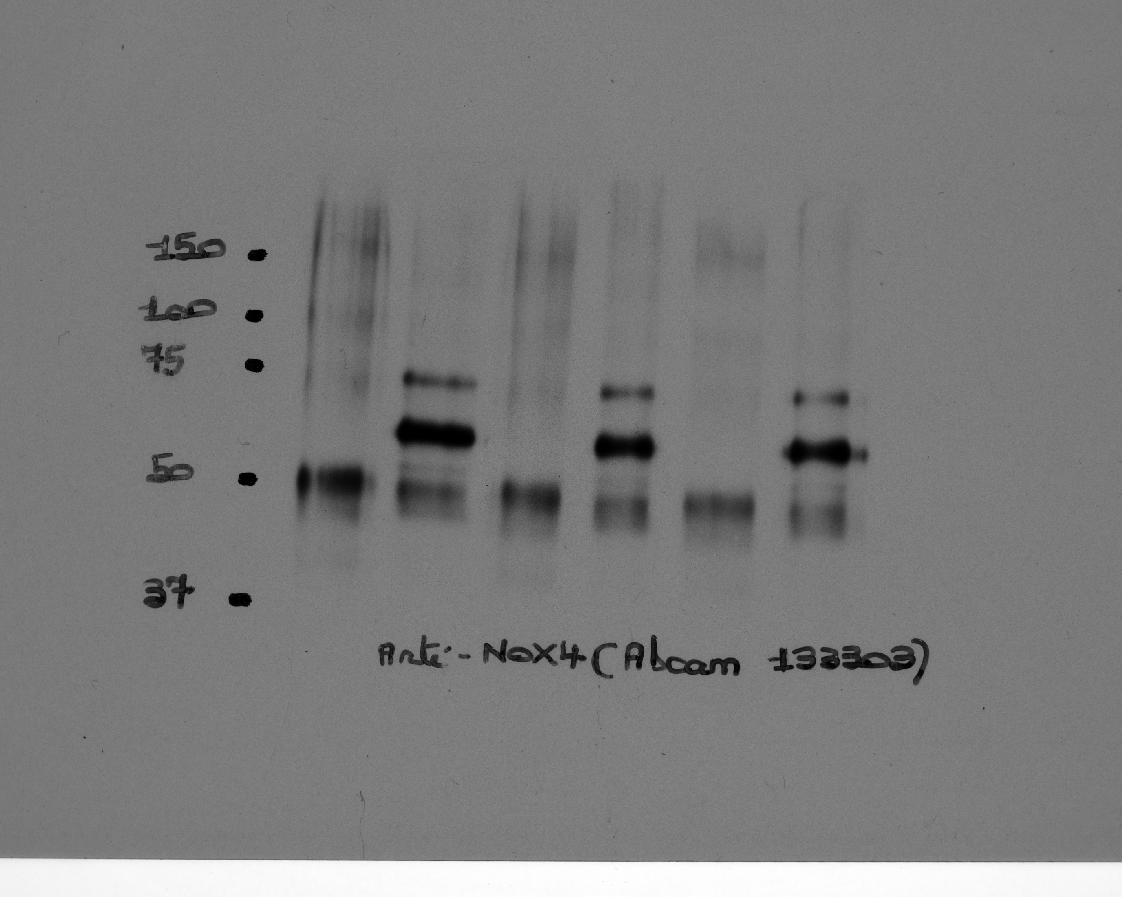

Supplement: Supplementary file 2 [file DataSheet2.ZIP › Original images for Western Blot/Figure 3/Figure 3 - NOX4 bis.tif]

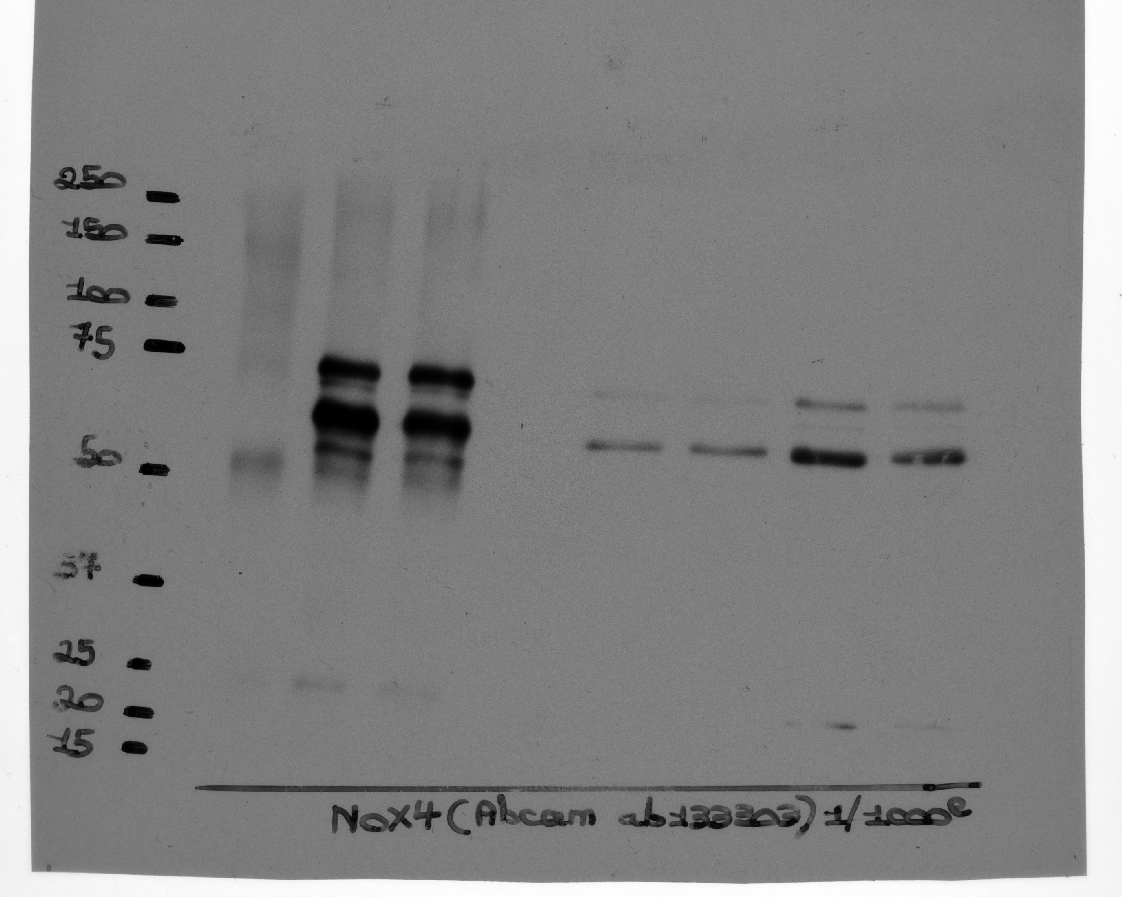

Supplement: Supplementary file 2 [file DataSheet2.ZIP › Original images for Western Blot/Figure 3/Figure 3 - NOX4.tif]

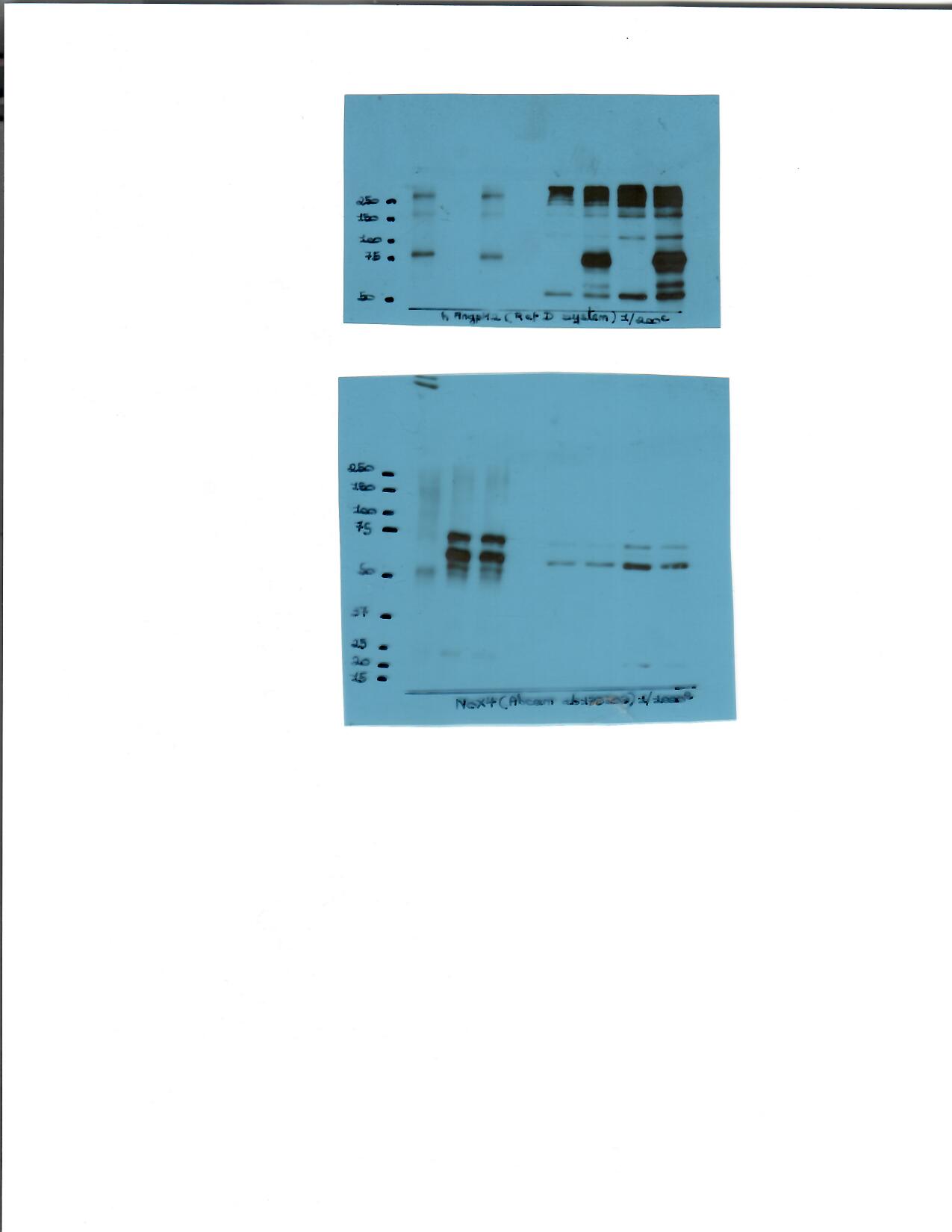

Supplement: Supplementary file 2 [file DataSheet2.ZIP › Original images for Western Blot/Figure 3/Figure 3 - X-ray film (1:2).jpg]

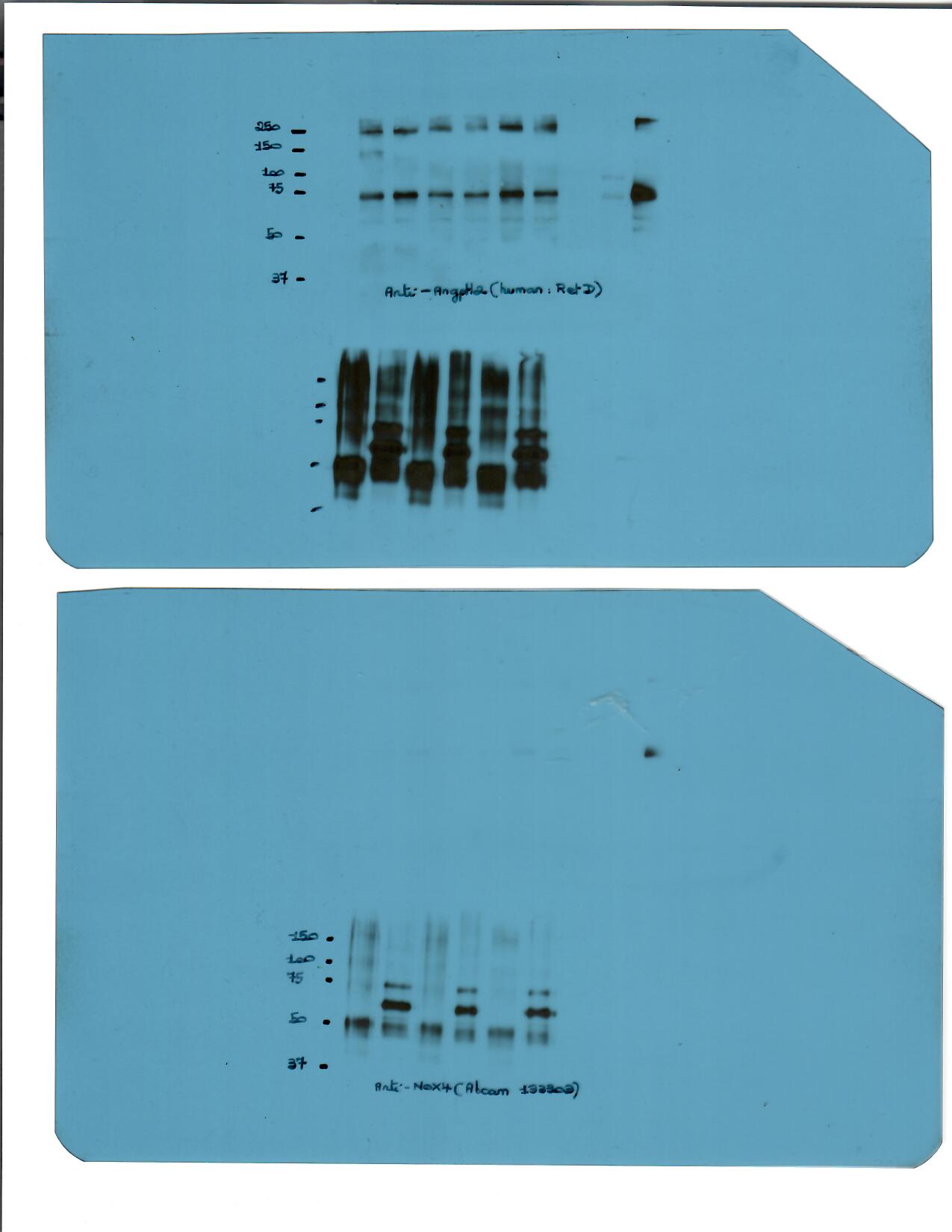

Supplement: Supplementary file 2 [file DataSheet2.ZIP › Original images for Western Blot/Figure 3/Figure 3 - X-ray film (2:2).jpg]

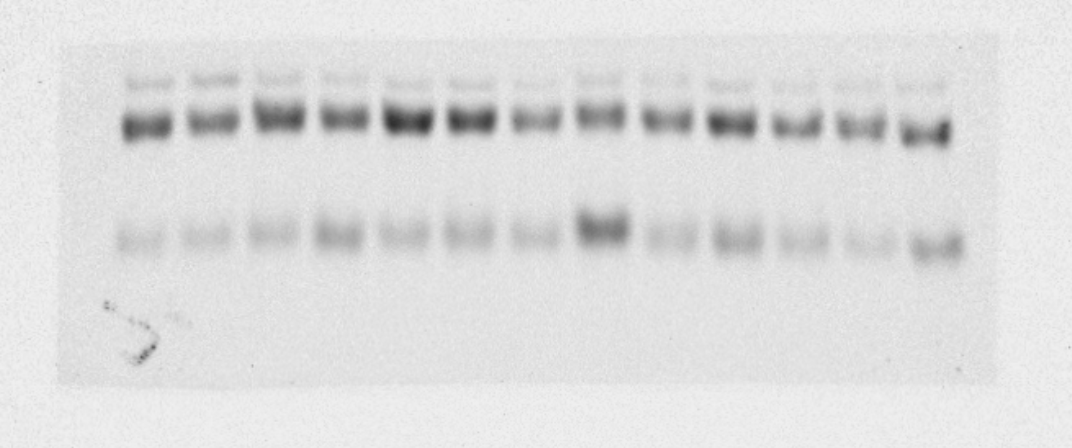

Supplement: Supplementary file 2 [file DataSheet2.ZIP › Original images for Western Blot/Figure 5/Figure 5A - GAPDH.tif]

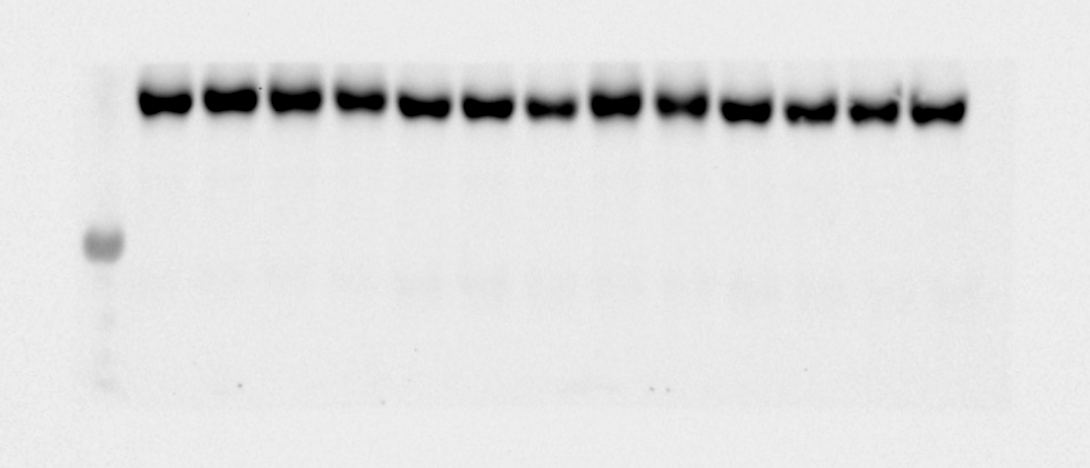

Supplement: Supplementary file 2 [file DataSheet2.ZIP › Original images for Western Blot/Figure 5/Figure 5A - p38 total.tif]

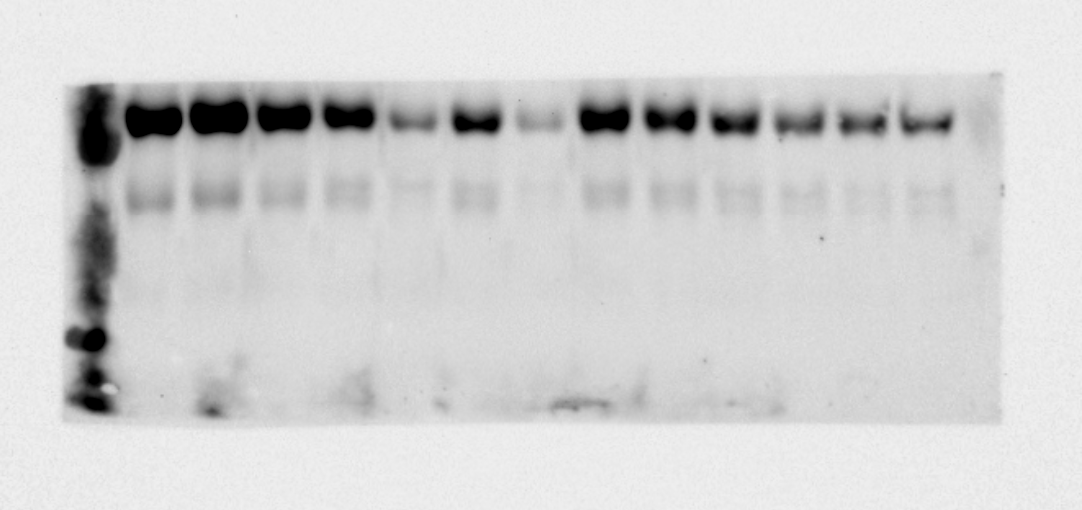

Supplement: Supplementary file 2 [file DataSheet2.ZIP › Original images for Western Blot/Figure 5/Figure 5A - phospho-p38.tif]

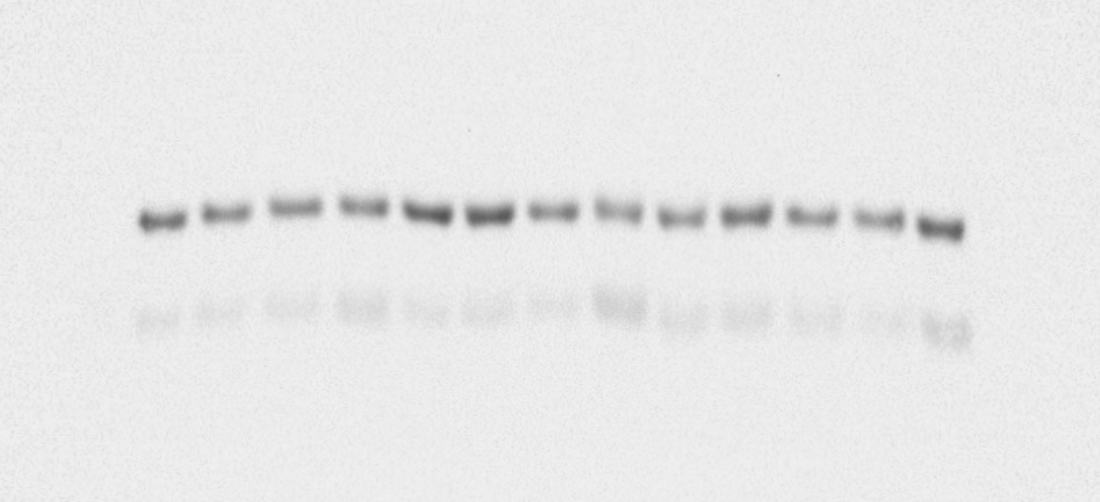

Supplement: Supplementary file 2 [file DataSheet2.ZIP › Original images for Western Blot/Figure 5/Figure 5B - GAPDH.tif]

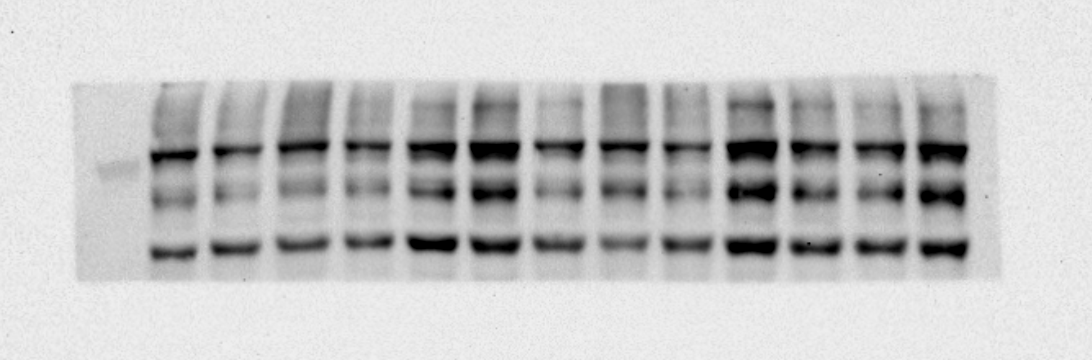

Supplement: Supplementary file 2 [file DataSheet2.ZIP › Original images for Western Blot/Figure 5/Figure 5B - NRF2.tif]

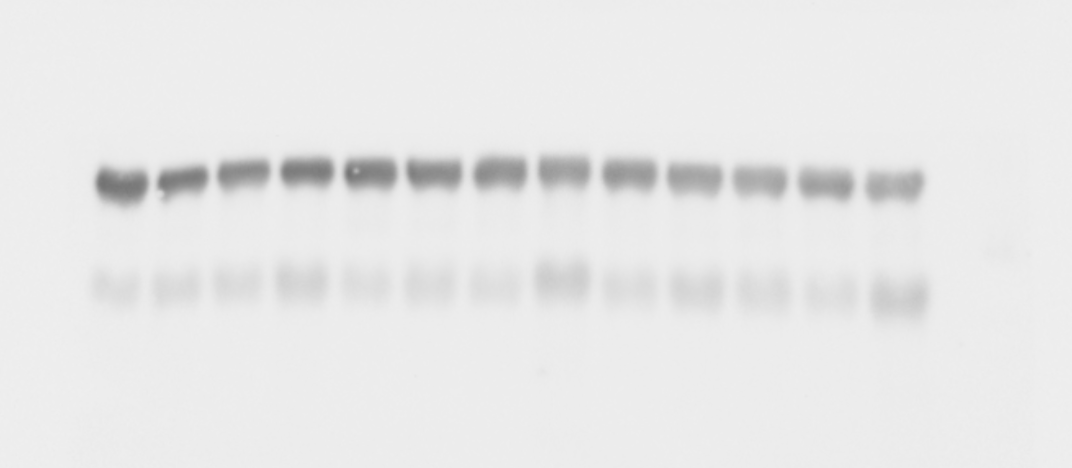

Supplement: Supplementary file 2 [file DataSheet2.ZIP › Original images for Western Blot/Figure 5/Figure 5C - GAPDH.tif]

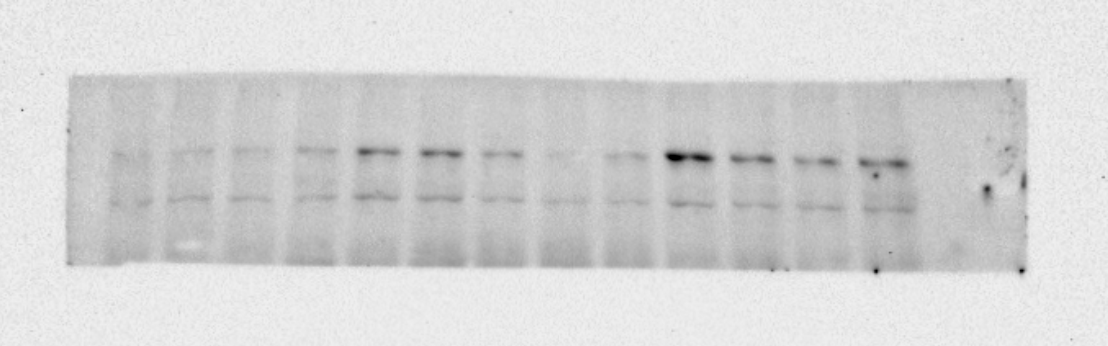

Supplement: Supplementary file 2 [file DataSheet2.ZIP › Original images for Western Blot/Figure 5/Figure 5C - Keap1.tif]

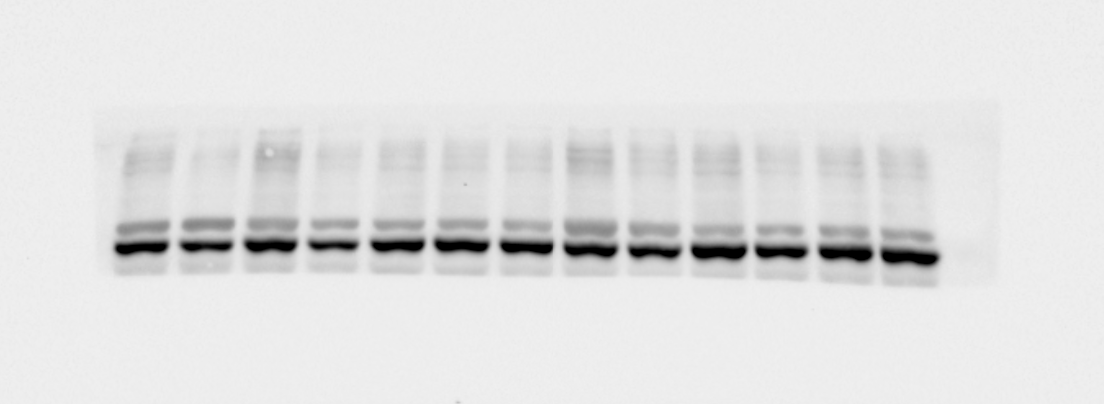

Supplement: Supplementary file 2 [file DataSheet2.ZIP › Original images for Western Blot/Figure 5/Figure 5D - Catalase.tif]

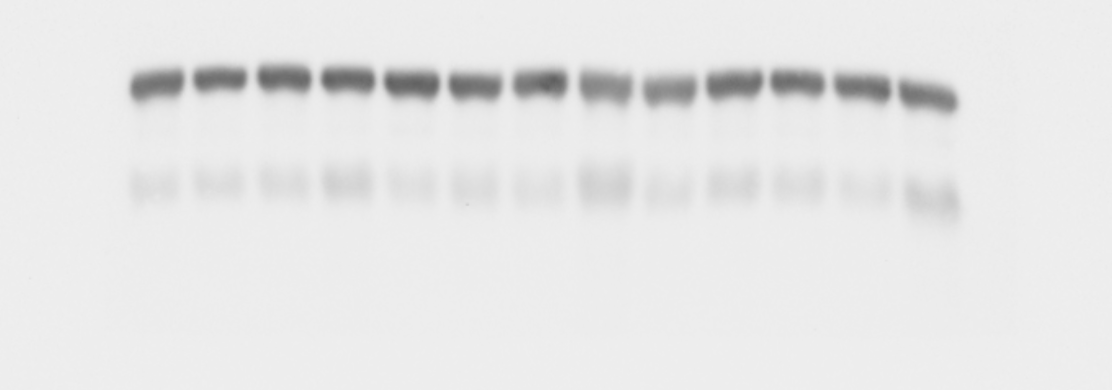

Supplement: Supplementary file 2 [file DataSheet2.ZIP › Original images for Western Blot/Figure 5/Figure 5D and E - GAPDH.tif]

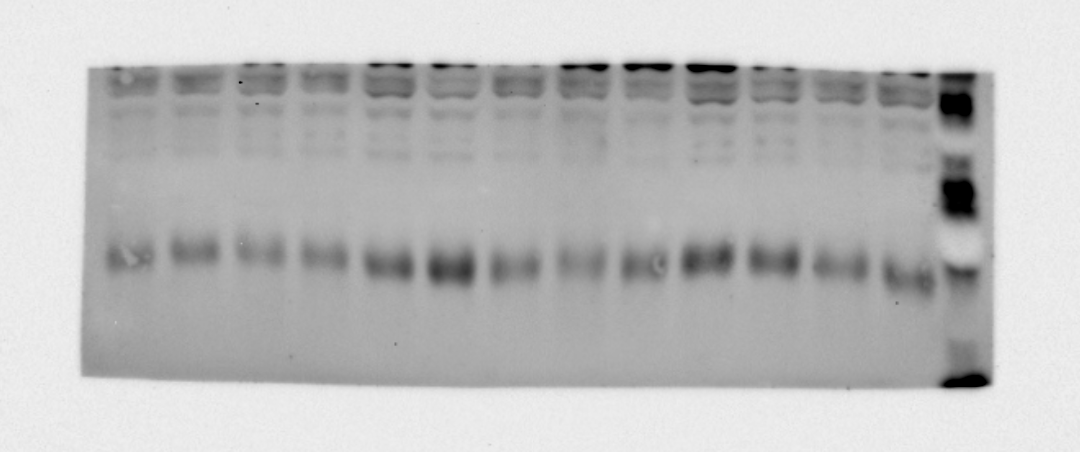

Supplement: Supplementary file 2 [file DataSheet2.ZIP › Original images for Western Blot/Figure 5/Figure 5E - p22phox.tif]

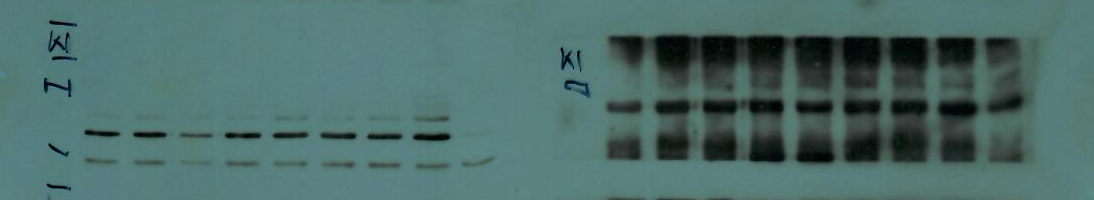

Supplement: Supplementary file 2 [file DataSheet2.ZIP › Original images for Western Blot/Figure S4/Figure S4 - NOX4 and GAPDH in kidney - crop.tif]

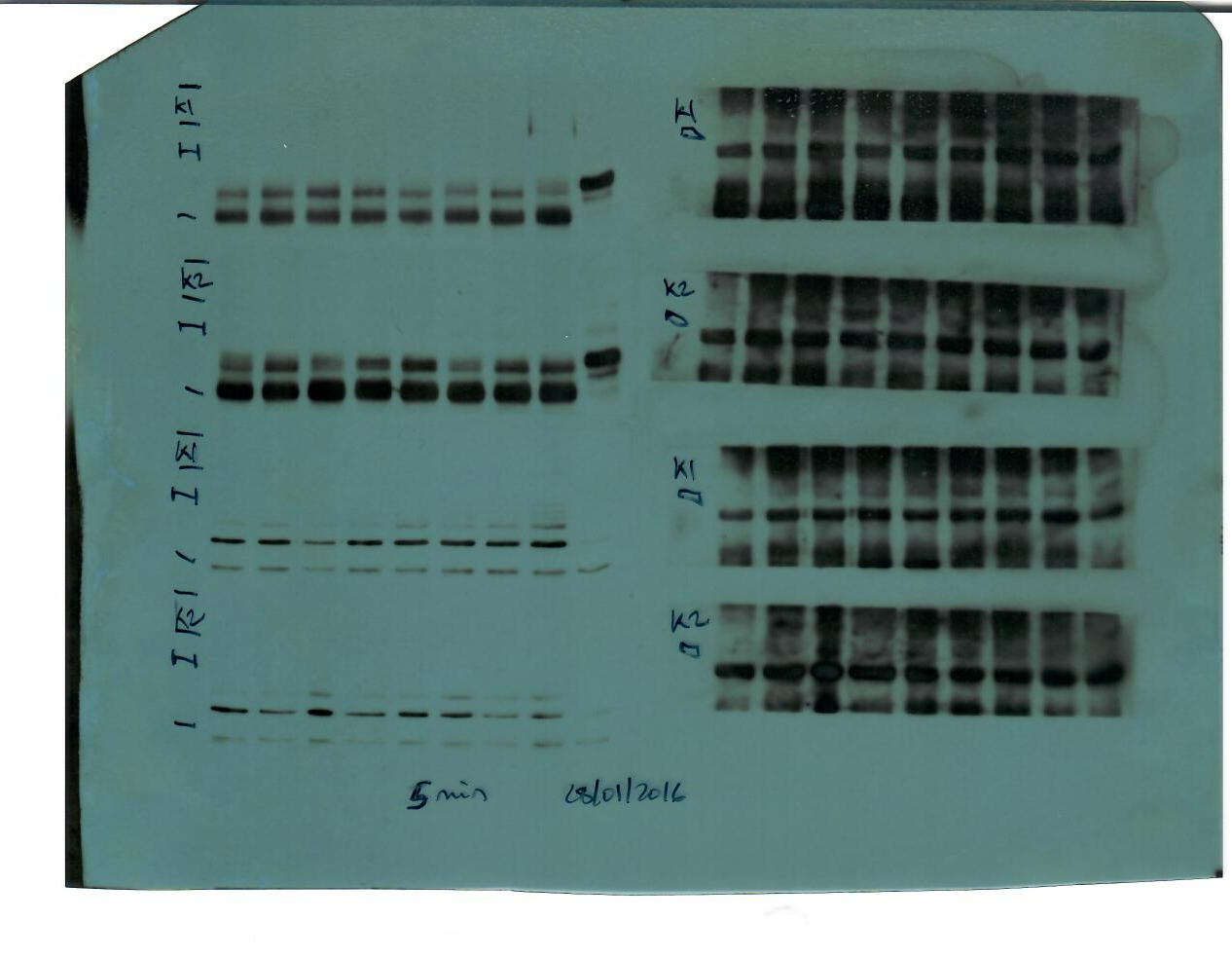

Supplement: Supplementary file 2 [file DataSheet2.ZIP › Original images for Western Blot/Figure S4/Figure S4 - NOX4 and GAPDH in kidney - whole X-ray film.tif]

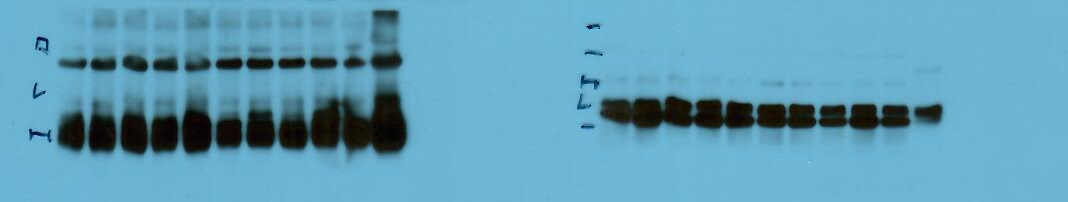

Supplement: Supplementary file 2 [file DataSheet2.ZIP › Original images for Western Blot/Figure S4/Figure S4 - NOX4 and GAPDH in live - crop.tif]

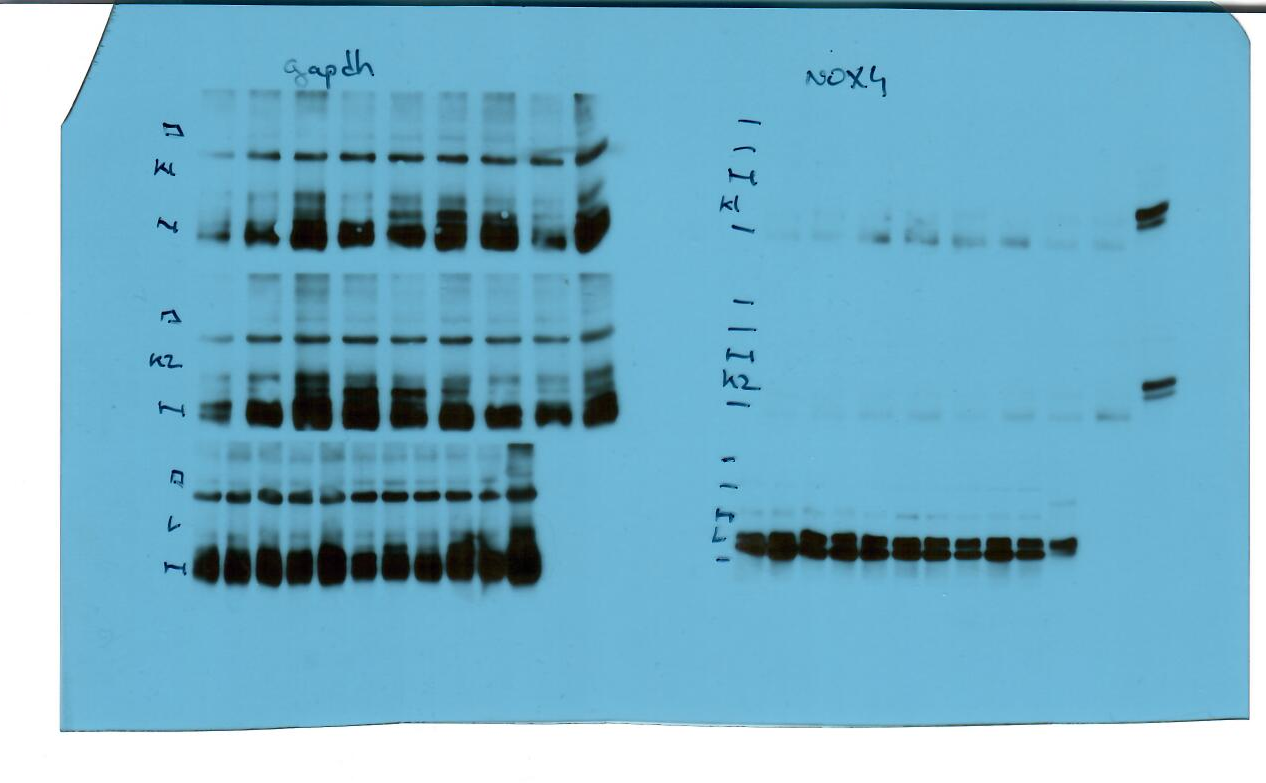

Supplement: Supplementary file 2 [file DataSheet2.ZIP › Original images for Western Blot/Figure S4/Figure S4 - NOX4 and GAPDH in live - whole X-ray film.tif]

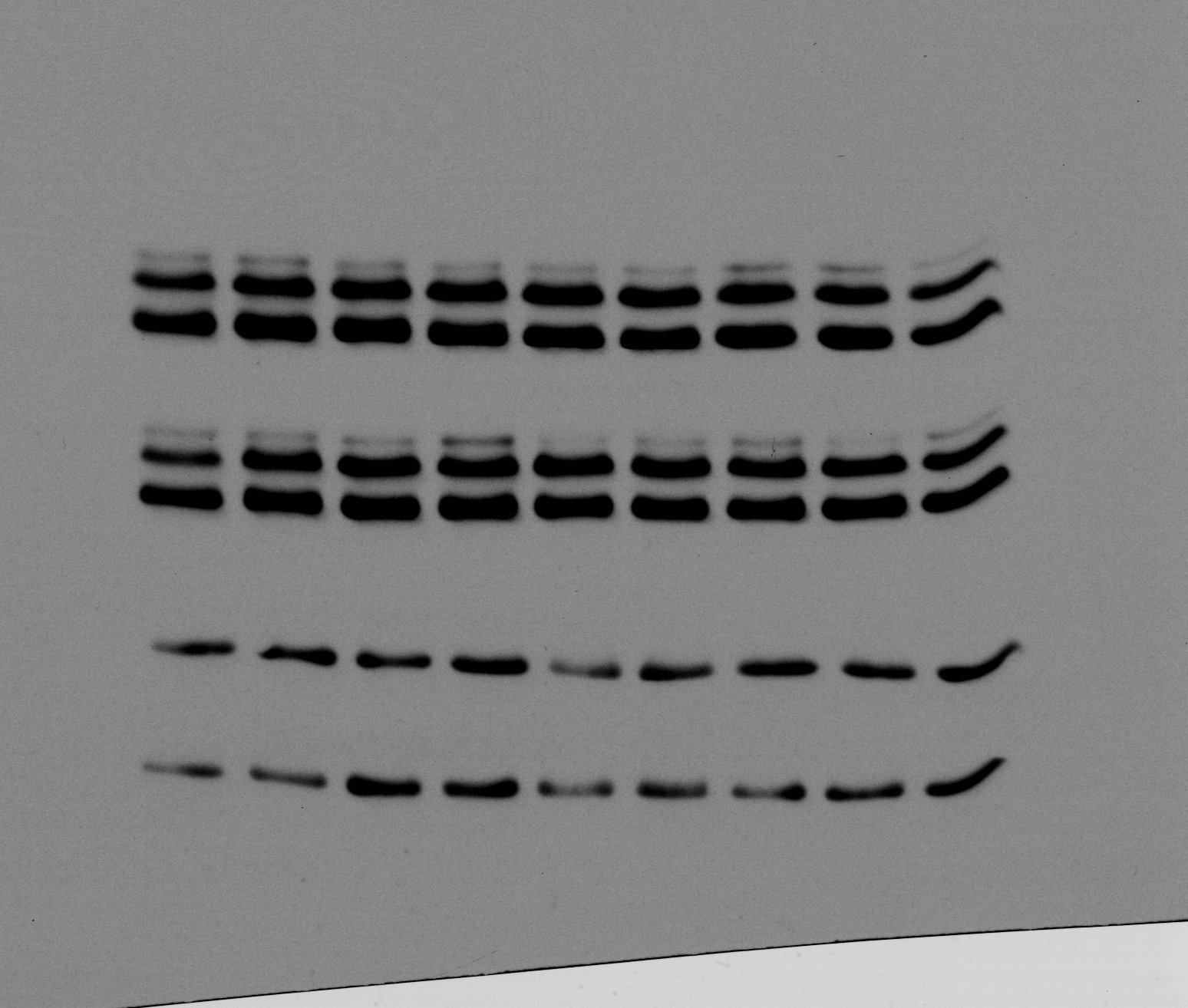

Supplement: Supplementary file 2 [file DataSheet2.ZIP › Original images for Western Blot/Figure S5/Figure S5 - GAPDH - crop.jpg]

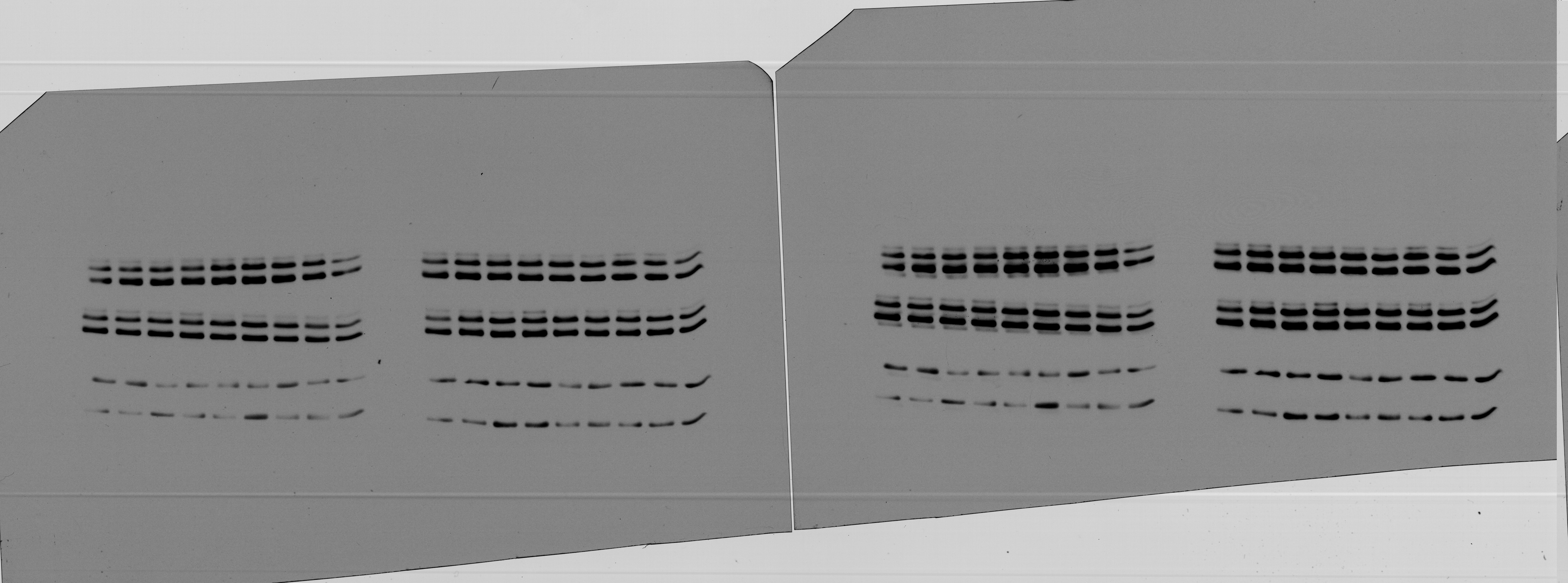

Supplement: Supplementary file 2 [file DataSheet2.ZIP › Original images for Western Blot/Figure S5/Figure S5 - GAPDH whole X-ray film.jpg]

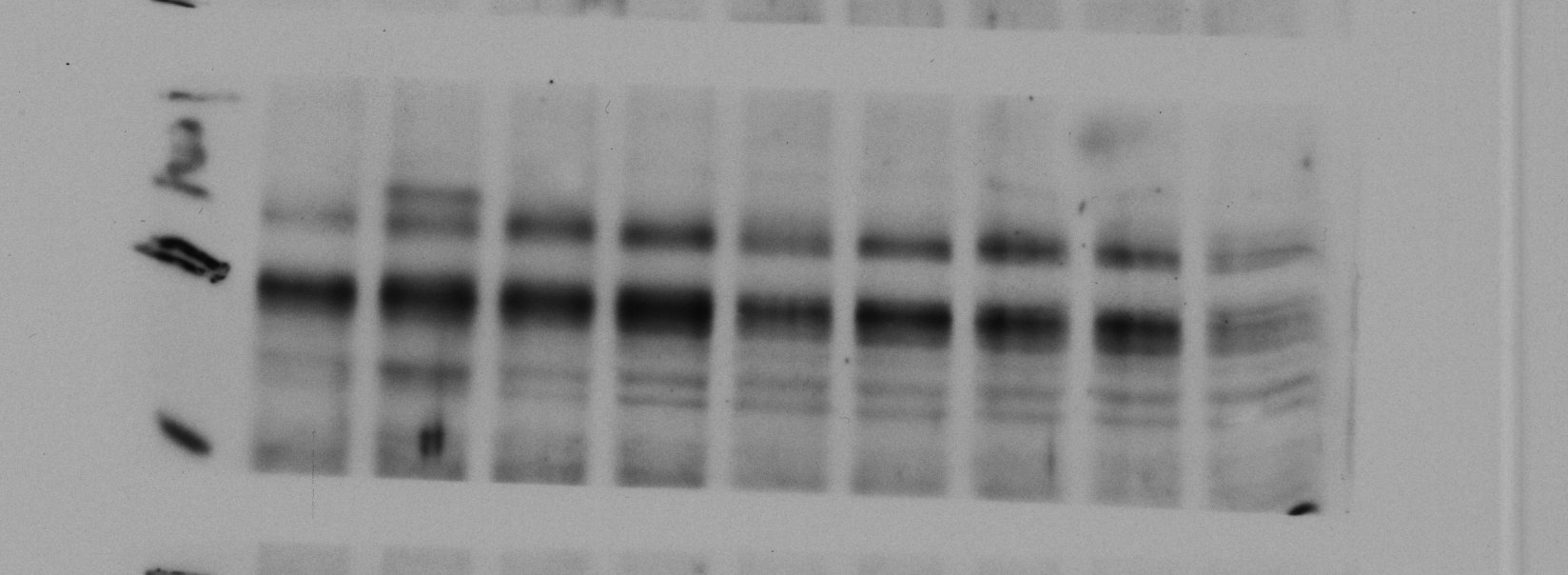

Supplement: Supplementary file 2 [file DataSheet2.ZIP › Original images for Western Blot/Figure S5/Figure S5 - NOX2 - crop.jpg]

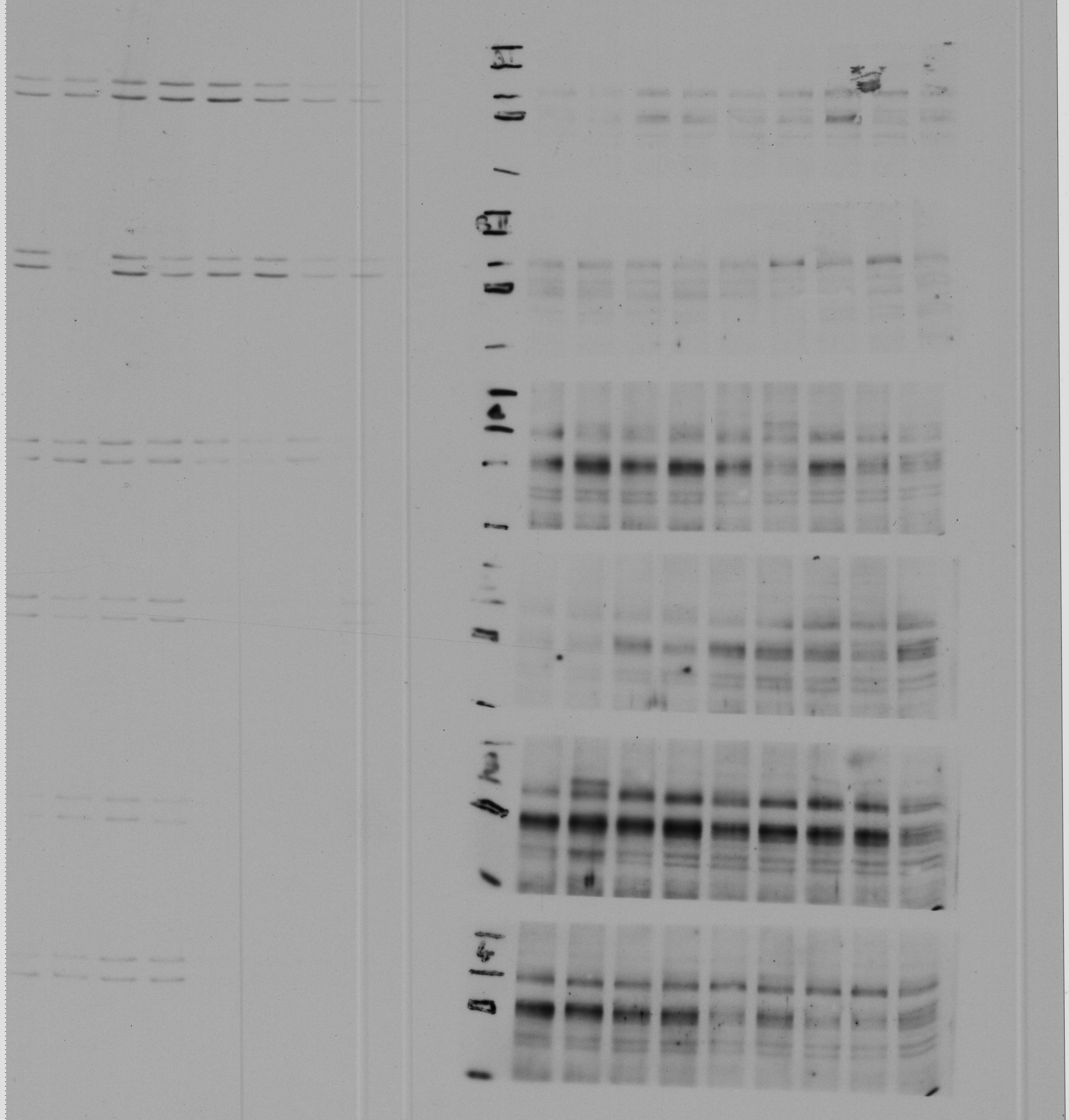

Supplement: Supplementary file 2 [file DataSheet2.ZIP › Original images for Western Blot/Figure S5/Figure S5 - NOX2 whole X-ray film.jpg]
